# Mixed-Substituted Single-Source Precursors for Si_1–*x*_Ge_*x*_ Thin Film Deposition

**DOI:** 10.1021/acs.inorgchem.2c02835

**Published:** 2022-10-19

**Authors:** Benedikt Köstler, Felix Jungwirth, Luisa Achenbach, Masiar Sistani, Michael Bolte, Hans-Wolfram Lerner, Philipp Albert, Matthias Wagner, Sven Barth

**Affiliations:** † Institute for Inorganic and Analytical Chemistry, Goethe University Frankfurt, Max-von-Laue-Str. 7, 60438 Frankfurt, Germany; ‡ Physical Institute, Goethe University Frankfurt, Max-von-Laue-Str. 1, 60438 Frankfurt, Germany; § Institute of Solid State Electronics, TU Wien, Gußhausstraße 25-25a, 1040 Vienna, Austria; ∥ Smart Materials, Evonik Operations GmbH, Untere Kanalstraße 3, 79618 Rheinfelden, Germany

## Abstract

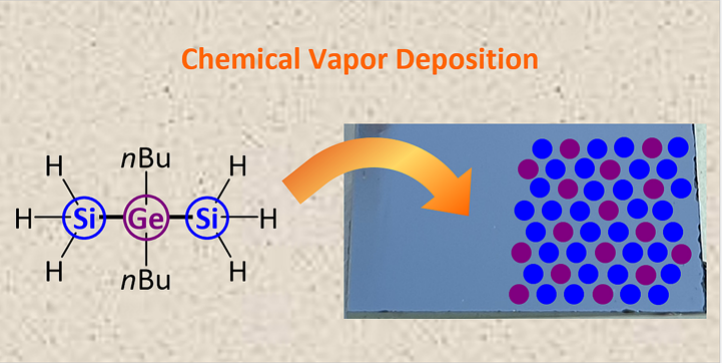

A series of new mixed-substituted heteronuclear precursors with preformed Si–Ge bonds has been synthesized via a two-step synthesis protocol. The molecular sources combine convenient handling with sufficient thermal lability to provide access to group IV alloys with low carbon content. Differences in the molecule–material conversion by chemical vapor deposition (CVD) techniques are described and traced back to the molecular design. This study illustrates the possibility of tailoring the physical and chemical properties of single-source precursors for their application in the CVD of Si_1–*x*_Ge_*x*_ coatings. Moreover, partial crystallization of the Si_1–*x*_Ge_*x*_ has been achieved by Ga metal-supported CVD growth, which demonstrated the potential of the presented precursor class for the synthesis of crystalline group IV alloys.

## Abstract 2

Three mixed-substituted molecular sources, (H_3_Si)_2_(GeR_2_)_*n*_ (with *n* = 1 or 2; R = Ph or *n*Bu), have been synthesized, which reveal high stability against oxidation. Their potential to act as single-source precursors for Si_1−*x*_Ge_*x*_ thin films by low-pressure chemical vapor deposition has been demonstrated. Retention of the Si:Ge ratio and low carbon contamination have been obtained in the material using a suitable precursor derivative.

## Introduction

Silicon–germanium Si_1–*x*_Ge_*x*_ thin films and nanostructures are extensively used in a large portfolio of applications including advanced transistors, quantum devices, photodetectors, electro-optical modulators, photovoltaics, microelectromechanical systems (MEMS), and thermoelectric generators.^1−7^ Moreover, Si_1–*x*_Ge_*x*_ interlayers can be used to control strain and defect densities in Si and Ge layers for electrical applications in CMOS device architectures.^8−10^

The group IV substitutional solid solution Si_1–*x*_Ge_*x*_ is often described as an alloy with complete solubility over the whole composition range, as illustrated in the binary phase diagram.^11^ However, the system has a strong segregation tendency and shows a large regime of coexistence of liquid and solid phases. Therefore, the solidification of a substitutional solid solution of a specific composition from the liquid phase is challenging. Typically, in situ formation of such materials well below the melting temperature is targeted to avoid large compositional variations within the Si_1–*x*_Ge_*x*_ crystals.

The most popular techniques for the controlled synthesis of thin layers and nanostructures of Si_1–*x*_Ge_*x*_ include molecular beam epitaxy using the elements as sources^12−14^ and the thermal decomposition of SiH_4_/GeH_4_ mixtures in chemical vapor deposition (CVD).^15,16^ In addition, alternative precursors for CVD synthesis such as higher silanes^17−20^ and dichlorosilane^21,22^ are reported. Crystal growth of Si_1–*x*_Ge_*x*_ on Si surfaces also includes the formation of nanodots accompanied by complex bulk and surface diffusion, leading to specific morphologies.^23^ This type of Stranski–Krastanov growth typically requires temperatures of ∼550–600 °C, and it can result in quite significant Si/Si_1–*x*_Ge_*x*_ intermixing at the interface. Therefore, lower substrate temperatures are typically targeted for the deposition of Si_1–*x*_Ge_*x*_ layers and surface-bound nanostructures on Si.

The electrical properties of the random Si_1–*x*_Ge_*x*_ alloy with cubic crystal phase have been summarized,^24^ but new developments will benefit from molecular precursors providing pre-formed Si–Ge building blocks. For instance, the growth of hexagonal Si_1–*x*_Ge_*x*_ was reported recently.^25^ The direct, tunable bandgap of this hexagonal polymorph should exhibit a narrower emission spectrum when the compositional variation within the Si_1–*x*_Ge_*x*_ is reduced. Such a very homogeneous atomic intermixing without segregation could be achieved with single-source precursors containing both Si and Ge in one molecule. Moreover, this single-source precursor concept should provide the best chances to enable growth of very recently proposed new polymorphs, providing access to direct bandgap Si_1–*x*_Ge_*x*_ materials differing in structure and bandgap^26^ or other metastable ternary materials with peculiar physical properties based on group IV elements.^27^

Single-source precursors carrying exclusively hydride ligands, such as H_3_SiGeH_3_ and Ge(SiH_3_)_4_, have been reported for the CVD of the Si_1–*x*_,Ge_*x*_ layers,^28^ but typical scrambling reactions during storage and inefficient synthesis strategies for their controlled formation are the most probable reasons why this strategy has not been further pursued. Moreover, the compounds are pyrophoric and require rigorous safety measures similar to the individual SiH_4_ and GeH_4_ sources.^29^

Very recently, a viable alternative to prepare mixed-substituted molecular Si–Ge precursors has been developed by the Wagner group.^30−32^ In these studies, the rich chemistry of the Si_2_Cl_6_/[*n*Bu_4_N]Cl system, which releases the powerful nucleophile [SiCl_3_]^−^ in situ, has been exploited for the facile formation of R_*n*_E–SiCl_3_ bonds (E = e.g., B, C, Si, Ge).^33^ For the thermal conversion of precursors to Si_1–*x*_Ge_*x*_, it should be noted that Si–C-containing silanes typically lead to silicon carbide,^34−37^ while Ge–C can be cleaved even at very moderate temperatures, yielding pure Ge material.^38−41^ Hence, the molecular design should consider these aforementioned tendencies and stability against inter- or intramolecular scrambling reactions during storage.

Here, we report on the synthesis and characterization of three mixed-substituted (H_3_Si)_2_(GeR_2_)_*n*_ (with *n* = 1 or 2; R = Ph or *n*Bu) molecular sources (**1-H**, **2-H**, and **3-H**) and their applicability as a new class of precursors for Si_1–*x*_Ge_*x*_ film formation by CVD. Important features are a tamed reactivity against oxidation and scrambling affinity by the introduction of Ge-alkyl/Ge-aryl moieties, while their design allows for an efficient alkyl cleavage by avoiding preformed Si–C bonds. The Si_1–*x*_Ge_*x*_ layers are characterized by X-ray diffraction (XRD), μ-Raman spectroscopy, energy dispersive X-ray spectroscopy (EDX), scanning electron microscopy (SEM), and atomic force microscopy (AFM).

## Results and Discussion

### New Mixed-Substituted Si–Ge Precursors

**Scheme 1:**
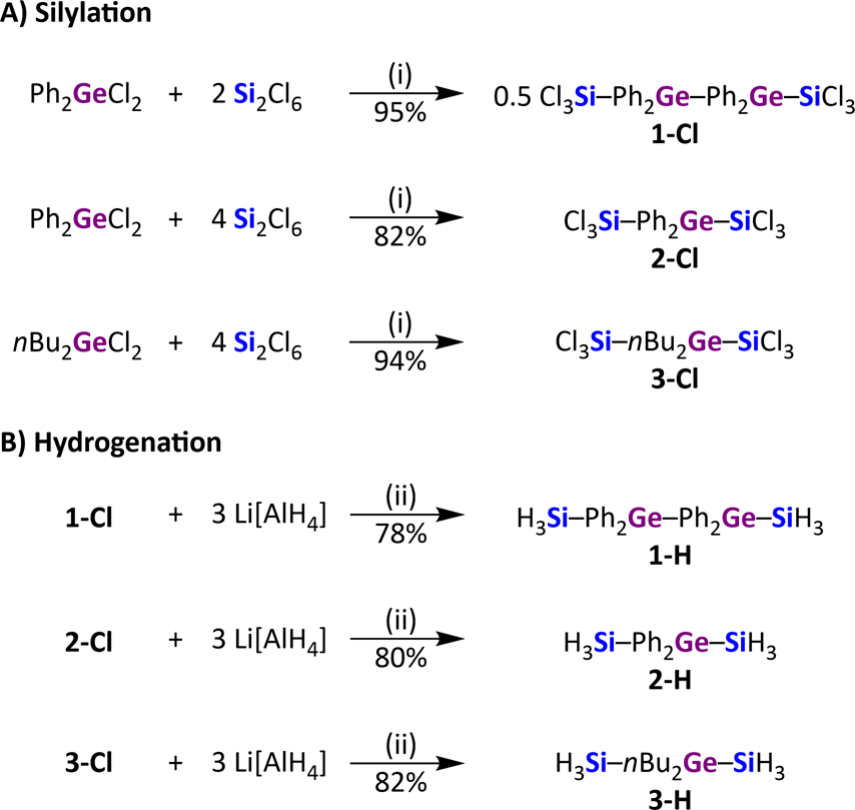
Syntheses of **1-Cl**, **2-Cl**, and **3-Cl** as well as Their Hydrogenation to **1-H**, **2-H**, and **3-H** (i) 0.2 equiv of [*n*Bu_4_N]Cl, CH_2_Cl_2_, rt. (ii) Et_2_O, rt.

The initial experiments targeting Cl_3_Si–Ph_2_Ge–SiCl_3_ (**2-Cl**; Scheme 1) were conducted by treatment of Ph_2_GeCl_2_ with 2 equiv of Si_2_Cl_6_ and 0.2 equiv of [*n*Bu_4_N]Cl as the most obvious stoichiometry for the formation of the desired compound. Surprisingly, the reaction led to the almost-quantitative formation of Cl_3_Si–Ph_2_Ge–Ph_2_Ge–SiCl_3_ (**1-Cl**), which was isolated as a colorless solid (95% yield). In this case, [SiCl_3_]^−^ apparently acted not only as a silylating agent but also as a reducing agent to establish the observed Ge–Ge bond. Subsequent screening of the Ph_2_GeCl_2_:Si_2_Cl_6_ stoichiometry revealed that an excess of Si_2_Cl_6_ (4 equiv) is in fact required to obtain Cl_3_Si–Ph_2_Ge–SiCl_3_ (**2-Cl**) as a colorless liquid in 82% yield. In an analogous reaction, the colorless, liquid alkyl derivative Cl_3_Si–*n*Bu_2_Ge–SiCl_3_ (**3-Cl**) was synthesized (94% yield; Scheme 1). A proposal of the formation process, which rationalizes the experimentally found stoichiometries, is detailed in the ESI (Figure S1).

Hydrogenation of **1-Cl**, **2-Cl**, or **3-Cl** with an excess of Li[AlH_4_] (3 equiv) resulted in quantitative conversions to **1-H**, **2-H**, and **3-H**. **1-H** was isolated as a colorless solid (78% yield), whereas **2-H** and **3-H** are colorless liquids (80 and 82% yields, respectively).

The solid-state structures of **1-Cl** and **1-H** were determined by single-crystal X-ray diffraction (Figure 1), showing indeed individual molecules without any sign of π–π interactions and the expected tetrahedral environment of the individual metalloid atoms. Additional information on crystal data and structure refinement are provided in Tables S1 and S2 of the SI. GC–MS data or elemental analyses as well as ^1^H, ^13^C{^1^H}, and ^29^Si NMR spectra are available for all compounds (Figures S2–S24) and described in the Experimental Section below. Particularly important information can be derived from the ^29^Si NMR signals of **1-H**, **2-H**, and **3-H**, which have chemical shifts in the range from −92.5 to −95.7 ppm and characteristic quartet multiplicities due to ^1^*J*(H,Si) coupling (192.3–198.7 Hz).

**Figure 1:**
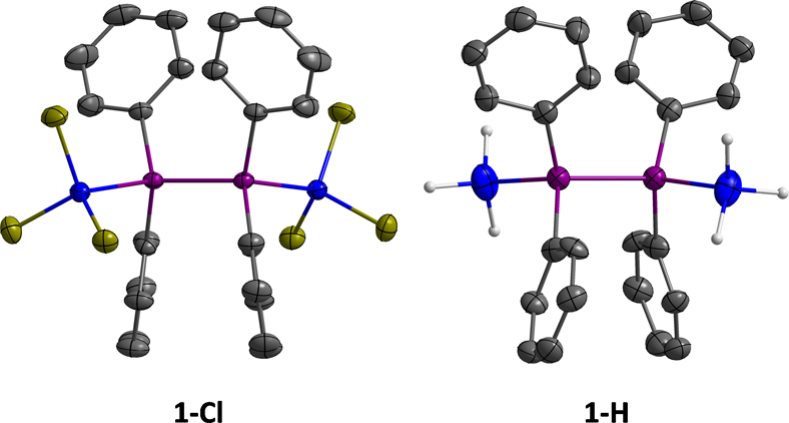
Solid-state structures of **1-Cl** and **1-H**, as determined by single-crystal X-ray diffraction (blue: Si; purple: Ge; yellow-green: Cl; dark gray: C; light gray: H; Ph–H atoms are omitted for clarity). Displacement ellipsoids are shown at the 50% probability level. Selected bond lengths [Å]: **1-Cl**: Ge–Ge = 2.4384(4), Ge–Si = 2.3855(6); **1-H**: Ge–Ge = 2.420(2), Ge–Si = 2.377(4).

Compounds **1-H**, **2-H**, and **3-H** have the structural arrangement targeted for the CVD of both Si_0.50_Ge_0.50_ and Si_0.67_Ge_0.33_ semiconductor layers. In addition, the molecules enable simple handling due to their inertness against oxidation/hydrolysis by introducing the organic ligands at the Ge atom(s) while avoiding Si–C bonding.

### Thin Film Deposition of Si_1–*x*_Ge_*x*_ by CVD

The volatilities of the precursors have been determined by heating the precursors under a reduced pressure of 10^–3^ mbar and collecting the volatiles. The H_3_Si–Ph_2_Ge–Ph_2_Ge–SiH_3_ precursor **1-H**, owning the highest molecular mass, is not volatile. The molecular source decomposes when heated up to 120 °C (10^–3^ mbar). Starting from ∼60 °C, fragments are liberated, and a highly viscous residue remains. Hence, **1-H** is not suitable for gas phase deposition by low-pressure CVD techniques.

In contrast, both **2-H** and **3-H** can be recondensed at moderate temperatures, with the *n*Bu derivative **3-H** being the most volatile. The most reasonable explanation for the increased volatility is a reduced molecular mass of 40 amu in the case of **3-H** and the absence of any intermolecular π–π interactions. The physical properties of the new Si–Ge precursors are summarized in Table 1.

**Table 1:** Volatility of Precursors Used for Material Synthesis and CVD Parameters Applied for Si_1–*x*_Ge_*x*_ Thin Film Deposition

	**1-H**	**2-H**	**3-H**
Si:Ge	1:1	2:1	2:1
recondensation (*p* ≈ 10^–3^ mbar)	decomp.	∼55–60 °C	∼20–25 °C
CVD		*T*_S_ ≈ 700 °C	*T*_S_ = 500–700 °C
(*p* < 10^–6^ mbar)		*T*_P_ > 25 °C	*T*_P_ > −20 to −5 °C

Low-pressure CVD was carried out in a home-built cold-wall reactor at a low background pressure of ∼10^–6^ mbar and adjusting the precursor temperature to provide sufficient vapor pressure for thin film growth. No carrier gas has been used in these studies, and the deposits’ composition will reflect the effectiveness of fragmentation channels in the absence of any reactive gases such as H_2_.

**Figure 2:**
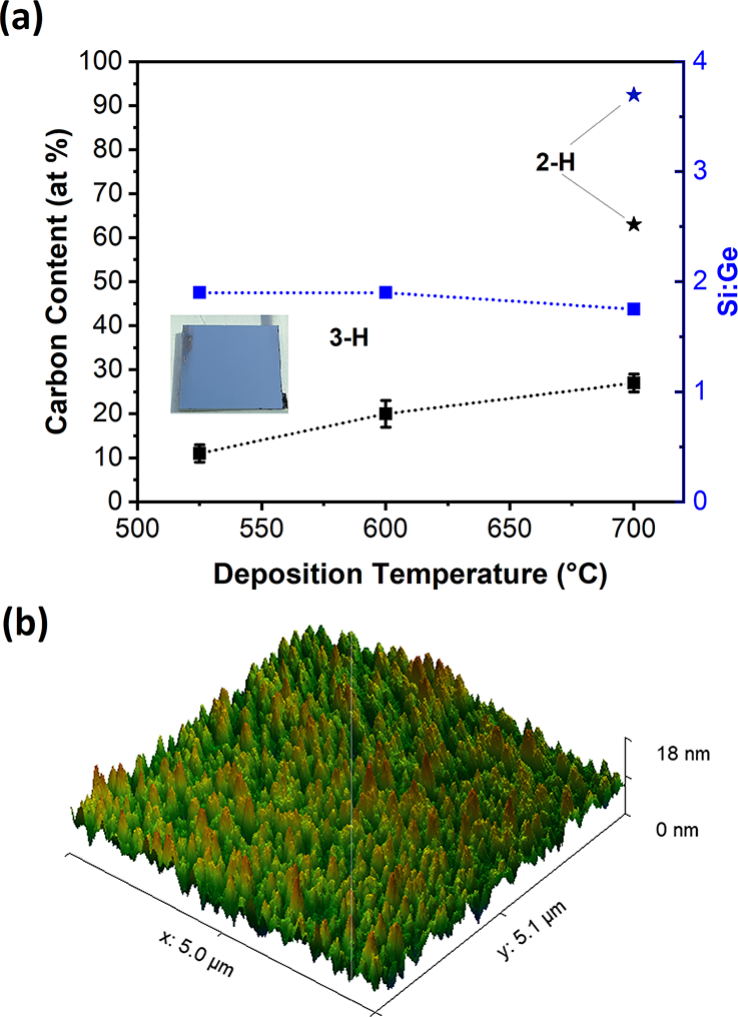
(a) Carbon content and Si:Ge ratio of CVD deposits determined by EDX analyses using **2-H** and **3-H** as precursors. The inset shows a silver-metallic deposit prepared using **3-H**. (b) The AFM image of a Si_1–*x*_Ge_*x*_ CVD coating at *T*_S_ = 525 °C using **3-H** shows the formation of a smooth film.

Controlled vaporization of the precursors required adjusting the precursor temperature to 0 to 25 °C for **2-H** and −20 to −5 °C for **3-H**. A substrate temperature (*T*_S_) sweep revealed a decomposition onset of *T*_S_ = ∼675 °C for **2-H**, while **3-H** leads to film formation at *T*_S_ = ∼500 °C. The actual film growth was carried out slightly above these onsets in order to achieve reasonable growth rates. Figure 2a shows the composition of Si_1–*x*_Ge_*x*_ films prepared by CVD on single-crystal sapphire substrates. The coating derived by using precursor **2-H** at *T*_S_ = 700 °C contains ∼60 at % C. Moreover, the Si:Ge ratio of 3.7:1 differs significantly from the 2:1 ratio in the precursor. A loss of Ge signifies a fragmentation liberating Ge-containing species from **2-H** and inefficiency of complete fragmentation.

In contrast, the decomposition onset of **3-H** is much lower at *T*_S_ = ∼500 °C. The lower decomposition temperature during CVD results in significantly reduced carbon contamination levels (11–27 at %) in the whole temperature range of *T*_S_ = 500–700 °C investigated for the growth of Si_0.67_Ge_0.33_ layers. The lower carbon incorporation illustrates an efficient fragmentation liberating the Ge-bound alkyl ligands even at the lowest temperatures. A likely explanation is β-hydride elimination, widely known in thermal decomposition of organometallic precursors,^42−44^ but the homolytic Ge–C bond cleavage or other reaction paths cannot be excluded at this point. This is in line with pure Ge deposition using *n*BuGeH_3_ as the precursor.^45^ In addition, inter- or intramolecular substituent exchange reaction between the silicon–germanium moieties should be largely diminished in order to achieve a low carbon content in thermal CVD. A preformed Si–C bond is not easily cleaved by thermal processing at moderate temperatures and typically leads to carbide-type deposits with C-contents depending on the fragmentation of the alkyl.^36,37,46^

CVD using **3-H** gives access to thin films with shiny silver metallic appearance (inset of Figure 2a). The Si:Ge ratio in the precursor is very close to the ideal 2:1 with values in the range of 1.9–1.7 according to EDX analyses. Representative EDX spectra used for the preparation of Figure 2a are provided in Figure S25. The CVD deposits using **3-H** are generally very smooth, and no significant features can be observed in SEM images. AFM provides more information, showing very smooth films with a root mean square (RMS) roughness of ∼2.24 nm for deposits from **3-H** at *T*_S_ = 525 °C (Figure 2b). The CVD films deposited at *T*_S_ = 700 °C are slightly rougher with an RMS of 14.97 nm (Figure S26).

**Figure 3:**
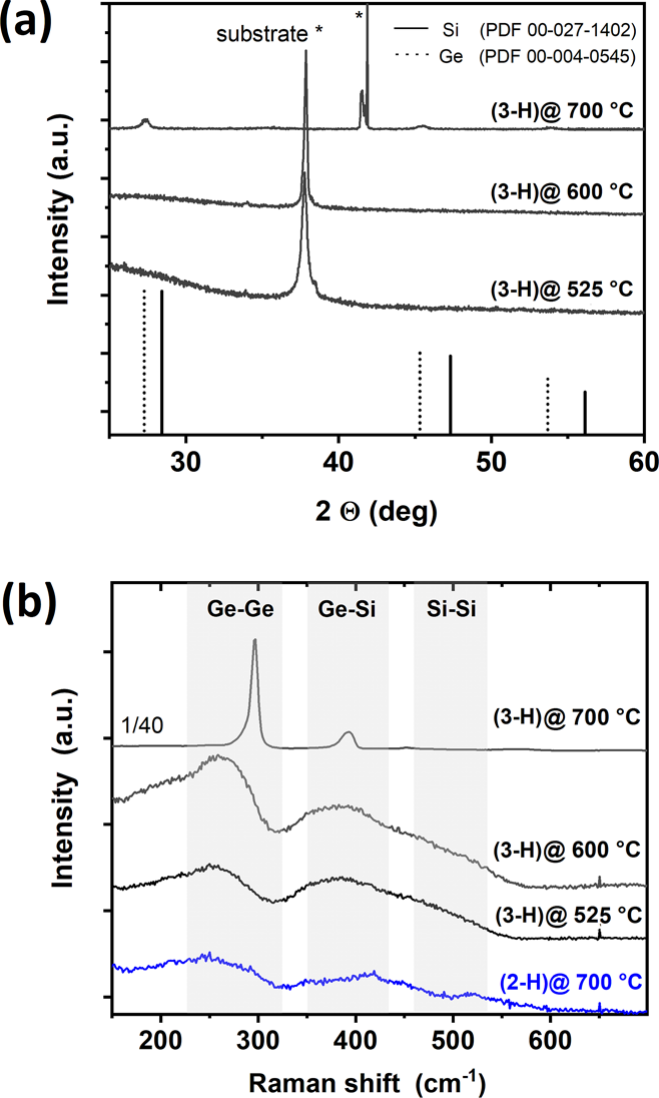
(a) XRD pattern of Si_1–*x*_Ge_*x*_ films prepared using **3-H** at substrate temperatures of 525–700 °C. The prominent reflections are assigned to r-cut sapphire (525 and 600 °C) and c-cut sapphire (700 °C). In addition, an unidentified additional reflection in the 700 °C film has been found at 41.5°. (b) Raman analysis reveals typical, broad peaks of amorphous Si_1–*x*_Ge_*x*_ films using **2-H** and **3-H**, while the coating using **3-H** at 700 °C shows a Ge–Ge signal close to the one expected for pure Ge, illustrating the Ge-rich Si_1–*x*_Ge_*x*_ phase.

In general, the **3-H**-derived CVD films are X-ray-amorphous in the moderate substrate temperature range of up to 600 °C as illustrated in Figure 3a. At the highest temperature of 700 °C, a broad reflection attributed to small nanoparticles of a Ge-rich Si_1–*x*_Ge_*x*_ phase can be observed. The predominantly amorphous nature of the CVD films could be a result of the carbon contamination delaying any onset of crystallization. Even deposits prepared using **3-H** at *T*_S_ = 525 °C containing only 11 at % C did not crystallize when post-growth annealing for 2 h at *T*_S_ = 700 °C was performed.

However, suitable information on the bonding in Si_1–*x*_Ge_*x*_ layers can be obtained from Raman analysis. Typically, three ranges of wavenumbers are considered for Ge–Ge, Ge–Si, and Si–Si with increasing values for the individual contributions. Figure 3b shows the typical broad signals for amorphous Si_1–*x*_Ge_*x*_ with *x* = 0.33.^47^ All Raman spectra are normalized and shifted vertically for clarity. A significant feature is the missing Si–Si peak in the Raman spectra expected in the range of ∼450–480 cm^–1^, which is typically weak in amorphous Si_1–*x*_Ge_*x*_ containing ∼33 at % Ge. The absence could be related to the carbon content within the samples reducing the Si–Si interactions. The Si–Ge Raman shift of ∼383 cm^–1^ for deposits of **3-H** grown at *T*_S_ = 500–600 °C is in the expected range according to the literature.^47^ The strong Raman signals for Si_1–*x*_Ge_*x*_ grown using **3-H** at 700 °C illustrate an onset of crystallization, but at the same time, the position of the Ge–Ge and Ge–Si signals indicate the formation of Ge-rich clusters. The most intense peak at 296 cm^–1^ is close to the pure Ge signal at 301 cm^–1^,^48^ while the Ge–Si peak is quite low in intensity and its position at ∼393 cm^–1^ is indicative of Si_1–*x*_Ge_*x*_ with a high Ge content.^49^ Since no information on strain is available, further discussion or calculation/determination of the actual composition of these Ge-rich nanocrystals within the otherwise amorphous matrix is not included.

**Figure 4:**
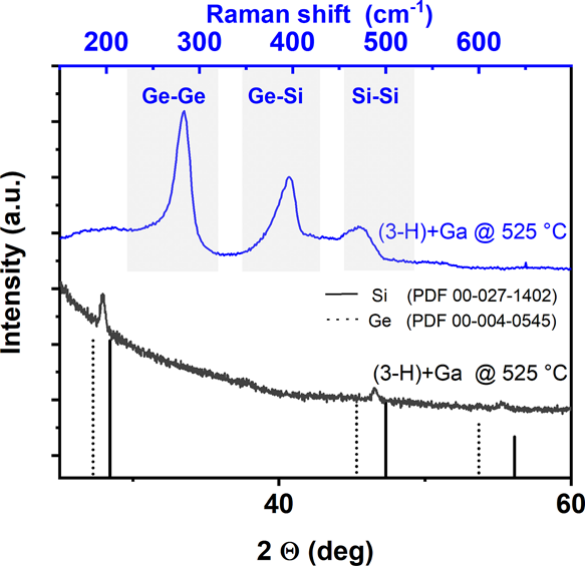
XRD pattern of Si_1–*x*_Ge_*x*_ films prepared using **3-H** at *T*_S_ = 525 °C using Ga as a crystallization enhancer. Clearly, a Si_1–*x*_Ge_*x*_ alloy with significant Si content has been obtained as indicated in the lower part of the graph. The Raman analysis in the upper section reveals all expected signals for Si_1–*x*_Ge_*x*_ obtained from the same sample used for XRD.

Crystallization at lower temperatures for the CVD of **3-H** has been attempted by the aid of an additional metal. No complete transformation is attempted but, rather, a partial crystallization of the deposit grown at *T*_S_ = ∼525 °C with ∼90 at % metalloid. For this purpose, tris-(dimethylamino)gallium(III) has been used for CVD of metallic Ga as a crystallization enhancer. We do not detail whether the Ga metal will support (i) the growth of the semiconductor by in situ metal-induced crystallization (MIC) of the amorphous Si_1–*x*_Ge_*x*_ deposit,^50,51^ (ii) a combined deposition of an amorphous Si_1–*x*_Ge_*x*_ layer forming simultaneously to a vapor–liquid–solid growth mode of the group IV alloy,^52,53^ or (iii) a combination of all the effects described in (i) and (ii). For these experiments, Ga has been pulsed in the CVD chamber prior to **3-H** or also three times in between the actual Si_1–*x*_Ge_*x*_ growth. Indeed, partial crystallization can be achieved at substrate temperatures of *T*_S_ = 525 °C as shown in Figure 4. According to calculations of the composition using Vegard’s law, the Si:Ge ratio in several deposits was between 1.1 and 1.7, while the overall Si:Ge ratio determined by EDX remained close to 2. Typical control samples for crystalline deposits are illustrated in Figure S27, showing the XRD pattern of a partially crystallized deposit with and without a Au/AuGa reference, which was sputtered on top of the Si_0.67_Ge_0.33_ film post-growth. Similarly, Raman spectroscopy in Figure 4 shows the three expected major peaks at ∼284, 396, and 496 cm^–1^, corresponding to first-order Ge–Ge, Si–Ge, and Si–Si optical phonon modes for crystalline Si_1–*x*_Ge_*x*_.^54^ A distinct difference to the phase-separated Ge-rich material obtained in CVD experiments at 700 °C is observed. Strong variations with predominantly crystalline and otherwise mostly amorphous material are recorded depending on the positioning in the μ-Raman measurement.

## Conclusions

A series of new Si–Ge-based precursors have been synthesized and tested in thermal conversion studies using CVD. Compounds **1-Cl**, **2-Cl**, and **3-Cl** were selectively synthesized from R_2_GeCl_2_, Si_2_Cl_6_, and Cl^–^ in good yields (R = Ph or *n*Bu). Subsequent hydrogenation of the SiCl_3_ groups with Li[AlH_4_] led to the quantitative formation of **1-H**, **2-H**, and **3-H**. Remarkably, these hydrogenated Si–Ge species are stable against exposure to air and moisture, which renders them safe for handling, storage, and transport.

This paper illustrates that alkyl-substituted H_3_Si–R_2_Ge–SiH_3_ such as **3-H** (R = *n*Bu) are suitable precursors for the CVD of inorganic coatings with predefined Si:Ge ratios. This reduces the parameters for the controlled synthesis of Si_1–*x*_Ge_*x*_ materials and, at the same time, should be key for the synthesis of highly homogeneous cubic Si_1–*x*_Ge_*x*_ alloys. The predominantly amorphous coatings retain the Si:Ge ratio reasonably well, and the contamination level of 10 at % C is low for CVD deposition without any carrier gas or complex procedures. Moreover, the Si_0.67_Ge_0.33_ material can be partially crystallized by the presence of metallic Ga, showing Si:Ge ratios in the crystals up to a value of 1.7. Hence, this report illustrates the first successful conversion of alkyl-modified single-source precursors to group IV alloys. A large variety of potential predefined compositions, including Si–Ge connectivities and modified ligand design, can be prepared by this precursor/synthesis strategy, providing a toolbox to be exploited for the synthesis of Si_1–*x*_Ge_*x*_ materials.

## Experimental Section

### General Considerations

All reactions were carried out under an inert-gas atmosphere (dry argon or nitrogen) using standard Schlenk or glove-box techniques. Commercially available starting materials were used as received: [*n*Bu_4_N]Cl (Sigma Aldrich), Li[AlH_4_] (Sigma Aldrich), *n*Bu_2_GeCl_2_ (Alfa Aesar), and Si_2_Cl_6_ (Evonik, see Acknowledgements). Ph_2_GeCl_2_ was prepared according to the literature.^55^*n*-Hexane, C_6_H_6_, and Et_2_O were dried over Na metal; CH_2_Cl_2_ was dried over CaH_2_. All solvents were freshly distilled prior to use. C_6_D_6_, [D_8_]THF, and CD_2_Cl_2_ were stored over molecular sieves (3 Å). NMR spectra were recorded at 298 K on a Bruker Avance III HD 500 spectrometer equipped with a Prodigy BBO 500 S1 probe. ^1^H/^13^C NMR spectra were referenced against (residual) solvent signals^56^ (C_6_D_6_: 7.16 ppm/128.06 ppm; [D_8_]THF: 1.72 ppm/25.31 ppm; CD_2_Cl_2_: 5.32 ppm/53.84 ppm). ^29^Si NMR spectra were calibrated against external Si(CH_3_**)**_4_ (δ(^29^Si) = 0). Abbreviations: s = singlet, t = triplet, q = quartet, m = multiplet. Resonance assignments were supported by two-dimensional NMR measurements (^1^H^13^C-HSQC, ^1^H^13^C-HMBC, ^1^H^29^Si-HSQC, and ^1^H^29^Si-HMBC). For the carbon atoms of the phenyl moieties, the resonance intensities were also considered. **Note:** The corresponding NMR spectra are shown in Figures S2–S24, together with numbering schemes for the specific C and H atom positions of each compound. For simplicity, only chemically inequivalent positions are labeled in these structural formulas.

GC–MS (gas chromatography–mass spectrometry) data were recorded using a Shimadzu GCMS-QP 2010SE. The stationary phase (Restek) had a length of 60 m with an inner diameter of 0.32 mm. The analyte was diluted with CH_2_Cl_2_ prior to the measurement. To avoid overloading the MS, a solvent cut was used. Samples were injected at 230 °C, and 1/10 thereof was transferred onto the column with a flow rate of 1.86 mL/min, carried by He gas. The oven was heated to 50 °C for 1 min; the temperature was subsequently elevated at a rate of 10 °C/min up to 250 °C and held for 40 min (60 min in the case of compound **1-H**). Finally, the oven temperature was elevated again at a rate of 25 °C/min up to 270 °C and held for 5 min. After a certain retention time τ, the substances exited the column and were ionized with 70 eV, and cationic fragments were measured within a range of *m*/*z* = 30–800 (mass per charges). Elemental analyses were performed at the Institute of Organic Chemistry and Chemical Biology, Goethe University Frankfurt, Germany.

### Synthesis of Cl_3_Si–Ph_2_Ge–Ph_2_Ge–SiCl_3_ (**1-Cl**)

A solution of [*n*Bu_4_N]Cl (0.933 g, 3.36 mmol) and Ph_2_GeCl_2_ (5.000 g, 16.79 mmol) in CH_2_Cl_2_ (40 mL) was prepared in a Schlenk tube. After addition of neat Si_2_Cl_6_ (9.030 g, 33.59 mmol) at room temperature, the reaction solution was stirred for 24 h. All volatiles were removed under reduced pressure to obtain a colorless solid, which was washed with CH_2_Cl_2_ (10 mL) to isolate **1-Cl** as a colorless solid. Yield: 5.810 g (8.041 mmol, 95%). Single crystals of **1-Cl** suitable for X-ray analysis were grown by slow evaporation of a solution in CH_2_Cl_2_:*n*-hexane (4:1).

^1^H NMR (500.2 MHz, CD_2_Cl_2_): δ = 7.54–7.51 (m, 8H; H-2), 7.46–7.42 (m, 4H; H-4), 7.39–7.35 (m, 8H; H-3); ^13^C{^1^H} NMR (125.8 MHz, CD_2_Cl_2_): δ = 136.4 (C-2), 132.2 (C-1), 130.5 (C-4), 129.4 (C-3); ^29^Si{^1^H} NMR (99.4 MHz, CD_2_Cl_2_): δ = 12.4; Elemental analysis: Calculated for C_24_H_20_Cl_6_Ge_2_Si_2_ (722.55): C 39.90; H 2.79. Found: C 40.64; H 3.02.

### Synthesis of Cl_3_Si–Ph_2_Ge–SiCl_3_ (**2-Cl**)

A solution of [*n*Bu_4_N]Cl (0.933 g, 3.36 mmol) and Ph_2_GeCl_2_ (5.000 g, 16.79 mmol) in CH_2_Cl_2_ (40 mL) was prepared in a Schlenk tube. After addition of neat Si_2_Cl_6_ (18.06 g, 67.18 mmol) at room temperature, the reaction solution was stirred for 1 h. All volatiles were removed under reduced pressure, and the highly viscous, colorless residue was extracted with *n*-hexane (50 mL). All volatiles were removed from the extract under reduced pressure to obtain **2-Cl** as a colorless liquid. Yield: 6.802 g (13.72 mmol, 82%).

^1^H NMR (500.2 MHz, CD_2_Cl_2_): δ = 7.65–7.62 (m, 4H; H-2), 7.53–7.46 (m, 6H; H-3 and H-4); ^13^C{^1^H} NMR (125.8 MHz, CD_2_Cl_2_): δ = 136.0 (C-2), 131.1 (C-4), 129.9 (C-3), 129.4 (C-1); ^29^Si{^1^H} NMR (99.4 MHz, CD_2_Cl_2_): δ = 9.7; Elemental analysis: Calculated for C_12_H_10_Cl_6_GeSi_2_ (495.71): C 29.08; H 2.03. Found: C 29.51; H 2.07.

### Synthesis of Cl_3_Si–*n*Bu_2_Ge–SiCl_3_ (**3-Cl**)

A solution of [*n*Bu_4_N]Cl (1.078 g, 3.879 mmol) and *n*Bu_2_GeCl_2_ (5.000 g, 19.40 mmol) in CH_2_Cl_2_ (60 mL) was prepared in a Schlenk tube. After addition of neat Si_2_Cl_6_ (20.86 g, 77.60 mmol) at room temperature, the reaction solution was stirred for 1 h. All volatiles were removed under reduced pressure, and the highly viscous, colorless residue was extracted with *n*-hexane (40 mL). All volatiles were removed from the extract under reduced pressure to obtain **3-Cl** as a colorless liquid. Yield: 8.280 g (18.17 mmol, 94%).

^1^H NMR (500.2 MHz, CD_2_Cl_2_): δ = 1.65–1.57 (m, 4H; H-2), 1.50–1.35 (m, 8H; H-1 and H-3), 0.93 (t, ^3^*J*(H,H) = 7.3 Hz, 6H; H-4); ^13^C{^1^H} NMR (125.8 MHz, CD_2_Cl_2_): δ = 28.7 (C-2), 26.6 (C-3), 13.6 (C-1 and C-4); ^29^Si{^1^H} NMR (99.4 MHz, CD_2_Cl_2_): δ = 13.5; GC–MS (EI): τ = 22.43 min, *m*/*z* = 399 ([M – *n*Bu]^+^), 343 ([M – 2 × *n*Bu]^+^), 321 ([M – SiCl_3_]^+^). All signals show the correct isotope pattern.

### Synthesis of Cl–Ph_2_Ge–Ph_2_Ge–Cl

A solution of [*n*Bu_4_N]Cl (0.093 g, 0.34 mmol) and Ph_2_GeCl_2_ (1.000 g, 3.359 mmol) in CH_2_Cl_2_ (10 mL) was prepared in a Schlenk tube. After addition of neat Si_2_Cl_6_ (0.455 g, 1.69 mmol) at room temperature, the reaction solution was stirred for 3 h. All volatiles were removed under reduced pressure to obtain a colorless solid (920 mg). Cl–Ph_2_Ge–Ph_2_Ge–Cl was identified as the main product by means of ^13^C NMR spectroscopy. We detected only minor Ph-containing impurities (plus [*n*Bu_4_N]Cl). No resonances were found in the ^29^Si NMR spectrum. The crude product was washed with CH_2_Cl_2_ (3 mL) to obtain Cl–Ph_2_Ge–Ph_2_Ge–Cl as a colorless solid. Yield: 0.379 g (0.722 mmol, 43%). Single crystals of Cl–Ph_2_Ge–Ph_2_Ge–Cl suitable for X-ray analysis were grown by slow evaporation of a solution in CH_2_Cl_2_:*n*-hexane (4:1).

The formation of Cl–Ph_2_Ge–Ph_2_Ge–Cl was unambiguously demonstrated by comparing the dimensions of its unit cell with those of the published solid state structure.^57^^1^H and ^13^C NMR chemical shifts were also identical to the reference values (C_6_D_6_).^58^

^1^H NMR (500.2 MHz, C_6_D_6_): δ = 7.77–7.73 (m, 8H; H-2), 7.02–7.05 (m, 12H; H-3 and H-4); ^1^H NMR (500.2 MHz, CD_2_Cl_2_): δ = 7.64–7.59 (m, 8H; H-2), 7.49–7.39 (m, 12H; H-3 and H-4); ^13^C{^1^H} NMR (125.8 MHz, C_6_D_6_): δ = 136.1 (C-1), 134.2 (C-2), 130.8 (C-4), 129.2 (C-3); ^13^C{^1^H} NMR (125.8 MHz, CD_2_Cl_2_): δ = 135.7 (C-1), 134.1 (C-2), 131.2 (C-4), 129.3 (C-3).

### Synthesis of H_3_Si–Ph_2_Ge–Ph_2_Ge–SiH_3_ (**1-H**)

A Schlenk tube was charged with **1-Cl** (2.100 g, 2.906 mmol) and Et_2_O (45 mL). Li[AlH_4_] (0.330 g, 8.70 mmol) was slowly added in portions of 50 mg at room temperature, and the reaction mixture was stirred for 2 h. All volatiles were removed under reduced pressure, and the residue was extracted with C_6_H_6_ (40 mL). H_2_O (1.0 mL) was carefully added to the extract at room temperature (moderate H_2_ evolution and precipitation of a colorless solid). The mixture was dried over Na_2_SO_4_ and filtered. All volatiles were removed from the filtrate under reduced pressure to obtain **1-H** as a colorless solid. Yield: 1.162 g (2.252 mmol, 78%). Single crystals of **1-H** suitable for X-ray analysis were grown by slow evaporation of a solution in CH_2_Cl_2_:*n*-hexane (4:1).

^1^H NMR (500.2 MHz, [D_8_]THF): δ = 7.42–7.36 (m, 8H; H-2), 7.31–7.22 (m, 12H; H-3 and H-4), 3.55 (s with satellites, ^1^*J*(H,Si) = 198.5 Hz, 6H; SiH_3_); ^13^C{^1^H} NMR (125.8 MHz, [D_8_]THF): δ = 137.4 (C-1), 136.2 (C-2), 129.5 (C-4), 129.2 (C-3); ^29^Si NMR (99.4 MHz, [D_8_]THF): δ = −93.8 (q, ^1^*J*(H,Si) = 198.5 Hz); GC–MS (EI): τ = 53.00 min, *m*/*z* = 516 ([M]^+^), 485 ([M – SiH_3_]^+^), 408 ([M – SiH_3_ – Ph]^+^), 259 ([Ph_2_Ge–SiH_3_]^+^). All signals show the correct isotope pattern.

### Synthesis of H_3_Si–Ph_2_Ge–SiH_3_ (**2-H**)

A Schlenk tube was charged with **2-Cl** (6.800 g, 13.72 mmol) and Et_2_O (70 mL). Li[AlH_4_] (1.562 g, 41.16 mmol) was slowly added in portions of 50 mg at room temperature, and the reaction mixture was stirred for 2 h. All volatiles were removed under reduced pressure, and the residue was extracted with *n*-hexane (40 mL). H_2_O (1.0 mL) was added to the extract at room temperature (moderate H_2_ evolution and precipitation of a colorless solid). The mixture was dried over Na_2_SO_4_ and filtered. All volatiles were removed from the filtrate under reduced pressure to obtain **2-H** as a colorless liquid. Yield: 3.160 g (10.93 mmol, 80%).

^1^H NMR (500.2 MHz, [D_8_]THF): δ = 7.50–7.46 (m, 4H; H-2), 7.34–7.30 (m, 6H; H-3 and H-4), 3.57 (s with satellites, ^1^*J*(H,Si) = 198.7 Hz, 6H; SiH_3_); ^13^C{^1^H} NMR (125.8 MHz, [D_8_]THF): δ = 137.0 (C-1), 135.8 (C-2), 129.5 (C-4), 129.4 (C-3); ^29^Si NMR (99.4 MHz, [D_8_]THF): δ = −92.5 (qq, ^1^*J*(H,Si) = 198.7 Hz, ^3^*J*(H,Si) = 2.9 Hz); GC–MS (EI): τ = 20.12 min, *m*/*z* = 258 ([M – SiH_3_]^+^), 213 ([M – Ph]^+^), 183 ([M – SiH_3_ – Ph]^+^). All signals show the correct isotope pattern.

### Synthesis of H_3_Si–*n*Bu_2_Ge–SiH_3_ (**3-H**)

A Schlenk tube was charged with **3-Cl** (8.000 g, 17.55 mmol) and Et_2_O (100 mL). Li[AlH_4_] (2.000 g, 52.70 mmol) was slowly added in portions of 50 mg at room temperature, and the reaction mixture was stirred for 24 h. All volatiles were removed under reduced pressure, and the residue was extracted with *n*-hexane (40 mL). H_2_O (1.0 mL) was added to the extract at room temperature (moderate H_2_ evolution and precipitation of a colorless solid). The mixture was dried over Na_2_SO_4_ and filtered. All volatiles were removed from the filtrate under reduced pressure to obtain **3-H** as a colorless liquid. Yield: 3.568 g (14.32 mmol, 82%).

^1^H NMR (500.2 MHz, [D_8_]THF): δ = 3.26 (s with satellites ^1^*J*(H,Si) = 192.3 Hz, 6H; SiH_3_), 1.50–1.43 (m, 4H; H-2), 1.40–1.31 (m, 4H; H-3), 1.18–1.12 (m, 4H; H-1), 0.90 (t, ^3^*J*(H,H) = 7.3 Hz, 6H; H-4); ^13^C{^1^H} NMR (125.8 MHz, [D_8_]THF): δ = 30.7 (C-2), 27.0 (C-3), 14.3 (C-1), 13.9 (C-4); ^29^Si NMR (99.4 MHz, [D_8_]THF): δ = −95.7 (qm, ^1^*J*(H,Si) = 192.3 Hz); GC–MS (EI): τ = 12.96 min, *m*/*z* = 250 ([M]^+^), 219 ([M – SiH_3_]^+^), 163 ([M – SiH_3_ – *n*Bu]^+^). All signals show the correct isotope pattern.

### CVD Process and Thin Film Characterization

CVD has been carried out in a home-built cold-wall reactor using high-frequency heating of a graphite or steel susceptor for indirect heating of sapphire (0001) or (11–20) (Crystal GmbH, Germany) and surface-oxidized Si (911) substrates with approx. 50 nm oxide (Crystec GmbH, Germany). The substrates are attached to the susceptor by silver paste to ensure efficient thermal contact. Substrate temperatures have been limited to *T*_S_ = 400–700 °C. The precursors were introduced in the reactor through a glass flange applying dynamic vacuum (∼10^–6^ mbar) while keeping the precursor temperatures in the range of −20 to 0 °C. Temperatures below 0 °C are maintained using a cooling bath based on chilled isopropyl alcohol as coolant. Typically, 40–80 mg of the precursors were used as source for the CVD experiments, and growth was carried out for 60–150 min. CVD based on tris-(dimethylamino)gallium(III) was carried out at *T*_S_ = 500 °C and a precursor temperature of 80 °C for 2 s per pulse using approx. 100 mg of the precursor to deposit metallic Ga. A similar CVD setup has been described in the literature for the growth of thin films and nanostructures using molecular sources.^59,60^

The sample composition was characterized by energy dispersive X-ray analysis (EDX) at a beam energy of 10 kV. Error bars represent variations between several EDX spectra recorded for at least three individual deposits using a defined set of parameters and several spots on the substrates. In addition, the standard-less quantification provides an estimate on the actual composition and will not be as accurate as EDX using defined material compositions for calibration. A slight overestimation of carbon content could be caused by additional carbon deposition during EDX associated to residual carbon sources in the background gas. Higher beam energies of 10 kV and thick deposits were used to limit the potential error determining the content of lighter elements. The topographical features of the deposits were determined by atomic force microscopy (AFM) operated in tapping mode (Nanosurf, Easyscan 2). For the sample characterization by X-ray diffraction, a Bruker D8 Discover was used in a Bragg–Brentano geometry. Match! software (Crystal Impact) was used for data analysis. μ-Raman measurements were performed on a WITec Alpha300 Raman system with a frequency-doubled Nd:YAG laser (λ = 532 nm) in a backscattering geometry. The power of the incident laser was adjusted to 250 μW. The laser was focused through an achromatic Nikon EPI EPlan 100× objective (NA = 0.9, WD = 0.23 mm), enabling a diffraction-limited spot size of ∼720 nm. The integration time was set to 300 s.

## Supporting Information Available

NMR spectra of the intermediates and hydrogenated precursors; important single crystal data of **1-Cl** and **1-H**; proposal of the formation process; additional EDX spectra; XRD, SEM, and AFM images of the CVD deposits (PDF)
