# Supplementary material for: Mixed-Substituted Single-Source Precursors for Si1–xGex Thin Film Deposition
Source: Inorg Chem. 2022 Oct 19;61(43):17248–55. doi: 10.1021/acs.inorgchem.2c02835 (PMC9627566; doi:10.1021/acs.inorgchem.2c02835)
Supplement: Supplementary file 1 — ic2c02835_si_001.pdf [file ic2c02835_si_001.pdf]

Supporting Information

Mixed-Substituted Single-Source Precursors for $Si_{1-x}Ge_x$ Thin Film Deposition

Benedikt Köstler,[1] Felix Jungwirth,[2] Luisa Achenbach,[1] Masiar Sistani,[3] Michael Bolte,[1] Hans-Wolfram Lerner,[1] Philipp Albert,[4] Matthias Wagner,[1,] and Sven Barth[2,]**

[1] Institute for Inorganic and Analytical Chemistry, Goethe University Frankfurt, Max-von-Laue-Str. 7, 60438 Frankfurt, Germany.

[2] Physical Institute, Goethe University Frankfurt, Max-von-Laue-Str. 1, 60438 Frankfurt, Germany.

[3] Institute of Solid State Electronics, TU Wien, Gußhausstraße 25-25a, 1040 Vienna, Austria.

[4] Evonik Operations GmbH, Smart Materials, Untere Kanalstraße 3, 79618 Rheinfelden, Germany.

Corresponding authors:

M. Wagner: matthias.wagner@chemie.uni-frankfurt.de

S. Barth: barth@physik.uni-frankfurt.de

Content:

1. Proposed Process for the Formation of the Si–Ge precursors S2
2. Single-Crystal X-ray Analyses S4
3. Plots of ^{1}H, ^{13}C{^{1}H}, ^{29}Si{^{1}H}, and ^{29}Si NMR Spectra S7
   3.1. NMR Spectra of **1-Cl** S7
   3.2. NMR Spectra of **2-Cl** S9
   3.3. NMR Spectra of **3-Cl** S11
   3.4. NMR Spectra of Cl–Ph_2Ge–Ph_2Ge–Cl S13
   3.5. NMR Spectra of **1-H** S15
   3.6. NMR Spectra of **2-H** S18
   3.7. NMR Spectra of **3-H** S21
4. Characterization of CVD Coatings S24
   4.1. General Considerations S24
5. References S27

1. Proposed Process for the Formation of the Si–Ge precursors

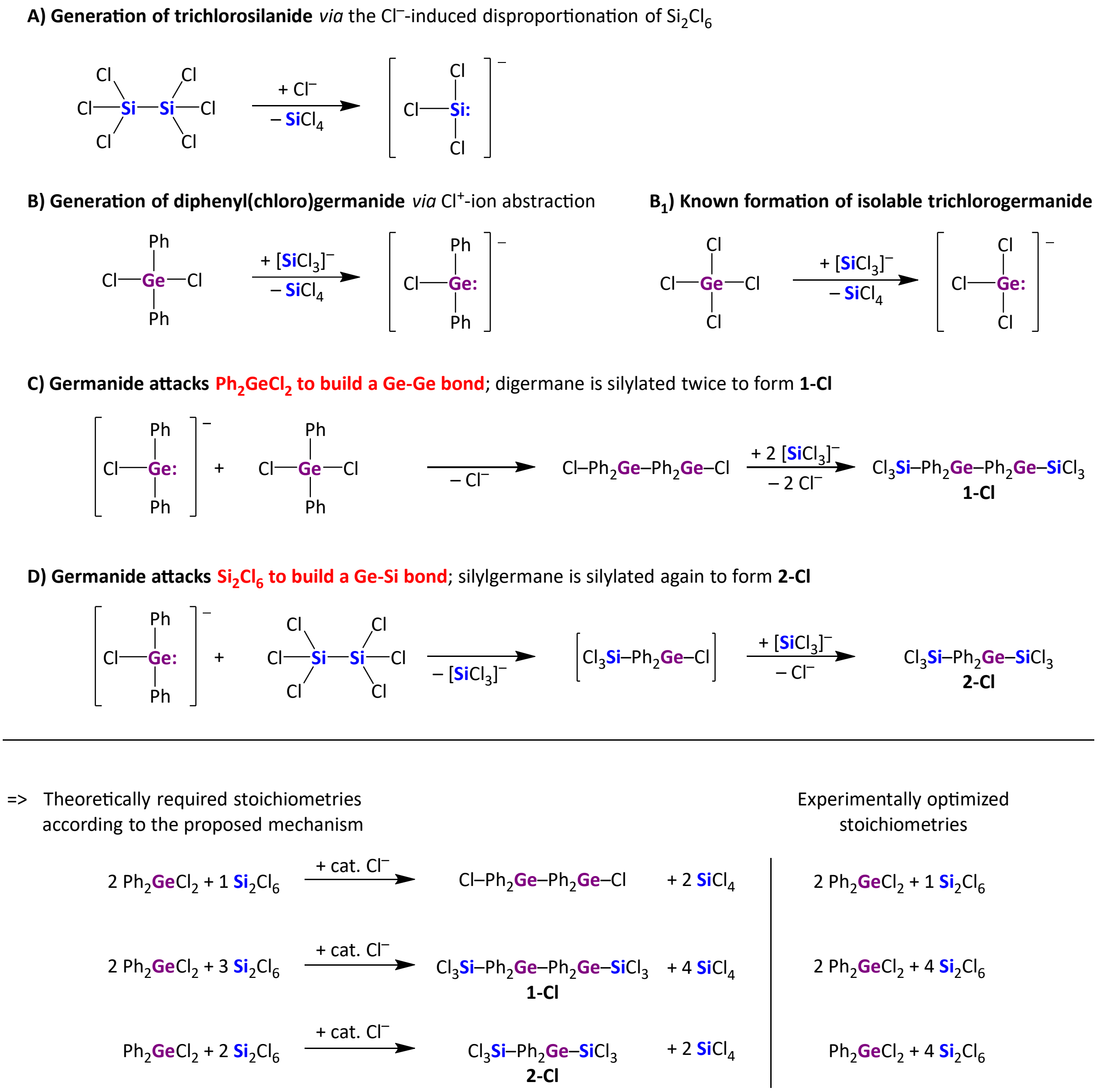

Figure S1. Conceivable mechanism to rationalize the stoichiometries *n*:*m* required for the formation of **1-Cl** and **2-Cl** from *n* Ph_2GeCl_2, *m* Si_2Cl_6, and cat. [*n*Bu_4N]Cl; for B_1, see Ref.[1]

A) The Cl^--induced heterolysis of the Si–Si bond in Si_2Cl_6 leads to the formation of $SiCl_4$ and the reactive intermediate $[SiCl_3]^-$.[2]

B) $[SiCl_3]^-$ abstracts a Cl^+ ion from Ph_2GeCl_2 to generate $[Ph_2GeCl]^-$ and $SiCl_4$. This assumption is supported by the known reaction of $GeCl_4$ with $[SiCl_3]^-$, which yields $SiCl_4$ and the isolable germanide $[GeCl_3]^-$ (cf. **B**$_1$).[1] Although $[GeCl_3]^-$ itself is a very weak nucleophile, its Ph derivatives have been successfully employed to generate digermanes, e.g., Ph_3GeNa +

Et_3GeBr → $Ph_3Ge–GeEt_3$ + NaBr.[3, 4] Given this background, it is reasonable to assume that also $[Ph_2GeCl]^-$ is sufficiently nucleophilic to attack either the Ge atom in Ph_2GeCl_2 (cf. **C**) or the Si atoms in Si_2Cl_6 or $SiCl_4$ (cf. **D**). These two pathways compete with each other.

C) We have shown by experiment that the reaction of 2 Ph_2GeCl_2 with 1 Si_2Cl_6 and cat. [$n$$Bu_4N$]Cl affords Cl–$Ph_2Ge$–$Ph_2Ge$–Cl as main product ($^{13}C\{^1H\}$ NMR spectroscopic control; isolated in 43% yield). Subsequent treatment of the digermane with 2 Si_2Cl_6/cat. Cl^- indeed furnished **1-Cl**, thereby confirming the proposed reaction mechanism. Although 1.5 equiv. Si_2Cl_6 should be sufficient for the one-pot reaction to **1-Cl**, in practice it is advantageous to use 2 equiv. Si_2Cl_6.

D) First, we have shown by experiment that **1-Cl** is inert toward $[SiCl_3]^-$ under the conditions applied in the synthesis of **2-Cl**. This rules out the digermane **1-Cl** as an intermediate along the path to **2-Cl**, but rather suggests that both Ge–Si bonds are formed already on the monogermane. According to our empirical findings, the theoretically required 2 equiv. Si_2Cl_6 are not sufficient for the formation of **2-Cl**, but rather give **1-Cl**. The problem is solved by use of 4 equiv. Si_2Cl_6 and we believe that the excess is necessary to ensure that Ge–Si-bond formation can outcompete the apparently very favorable Ge–Ge-bond formation.

2. Single-Crystal X-ray Analyses

Data sets for both structures were collected on a STOE IPDS II two-circle diffractometer with a Genix Microfocus tube with mirror optics using MoK_α radiation (λ = 0.71073 Å). The data sets were scaled using the frame-scaling procedure in the *X-AREA* program system.[5] The structures were solved by direct methods using the program *SHELXS*[6] and refined against F^2 with full-matrix least-squares techniques using the program *SHELXL*[6].

CCDC files CCDC 2182827 (**1-Cl**) and CCDC 2182828 (**1-H**) contain the supplementary crystallographic data for this paper and can be obtained free of charge from The Cambridge Crystallographic Data Centre via www.ccdc.cam.ac.uk/data_request/cif.

1-H crystallizes with two half molecules in the asymmetric unit. The other half of both molecules is generated by a two-fold rotation axis. One molecule is shown in Figure 1 of the main article. In the second molecule, the Ge atom and one SiH_3 group are disordered over two equally occupied positions. The absolute structure was determined, Flack-x-parameter 0.06(4).

Table S1. Crystal data and structure refinement for **1-Cl**.

CCDC code	2182827	
Empirical formula	$C_{24} H_{20} Cl_6 Ge_2 Si_2$	
Formula weight	722.46	
Temperature	173(2) K	
Wavelength	0.71073 Å	
Crystal system	Monoclinic	
Space group	*C2/c*	
Unit cell dimensions	a = 9.8305(5) Å	α = 90°
	b = 17.8639(7) Å	β = 101.309(4)°
	c = 16.8410(9) Å	γ = 90°
Volume	2900.0(2) $Å^3$	
Z	4	
Density (calculated)	1.655 Mg/m^3	
Absorption coefficient	2.722 mm^{-1}	
F(000)	1432	
Crystal size	0.250 × 0.200 × 0.200 mm^3	
Theta range for data collection	3.729 to 33.205°	
Index ranges	–15<=h<=15, –27<=k<=27, –25<=l<=24	
Reflections collected	33798	
Independent reflections	5510 [R(int) = 0.0864]	
Completeness to theta = 25.000°	99.2 %	
Absorption correction	Semi-empirical from equivalents	
Max. and min. transmission	1.000 and 0.598	
Refinement method	Full-matrix least-squares on F^2	
Data / restraints / parameters	5510 / 0 / 154	
Goodness-of-fit on F^2	0.981	
Final R indices [I>2sigma(I)]	$R1$ = 0.0409, $wR2$ = 0.1014	
R indices (all data)	$R1$ = 0.0475, $wR2$ = 0.1060	
Largest diff. peak and hole	1.333 and –0.643 e·$Å^{-3}$	

Symmetry transformation used to generate equivalent atoms:–x+1, y, –z+3/2

Table S2. Crystal data and structure refinement for **1-H**.

CCDC code	2182828	
Empirical formula	$C_{24} H_{26} Ge_2 Si_2$	
Formula weight	515.81	
Temperature	173(2) K	
Wavelength	0.71073 Å	
Crystal system	Monoclinic	
Space group	*C2*	
Unit cell dimensions	a = 16.771(4) Å	α = 90°
	b = 11.529(2) Å	β = 110.932(18)°
	c = 13.476(3) Å	γ = 90°
Volume	2433.7(9) $Å^3$	
Z	4	
Density (calculated)	1.408 Mg/m^3	
Absorption coefficient	2.576 mm^{-1}	
F(000)	1048	
Crystal size	0.180 × 0.150 × 0.120 mm^3	
Theta range for data collection	3.351 to 26.440°	
Index ranges	–20<=h<=20, –14<=k<=14, –16<=l<=16	
Reflections collected	12091	
Independent reflections	4965 [R(int) = 0.0747]	
Completeness to theta = 25.000°	99.6 %	
Absorption correction	Semi-empirical from equivalents	
Max. and min. transmission	1.000 and 0.750	
Refinement method	Full-matrix least-squares on F^2	
Data / restraints / parameters	4965 / 37 / 267	
Goodness-of-fit on F^2	1.077	
Final R indices [I>2sigma(I)]	$R1$ = 0.0842, $wR2$ = 0.2128	
R indices (all data)	$R1$ = 0.0898, $wR2$ = 0.2174	
Largest diff. peak and hole	1.112 and –1.034 e·$Å^{-3}$	

Symmetry transformation used to generate equivalent atoms: –x+1, y+2, –z+1

3. Plots of ^{1}H, $^{13}C\{^{1}H\}$, $^{29}Si\{^{1}H\}$, and ^{29}Si NMR Spectra

3.1. NMR Spectra of 1-Cl

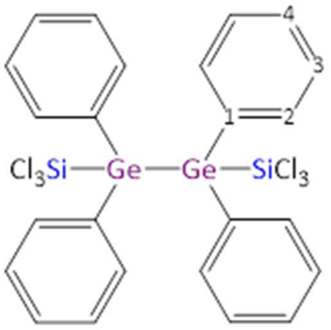

1-Cl

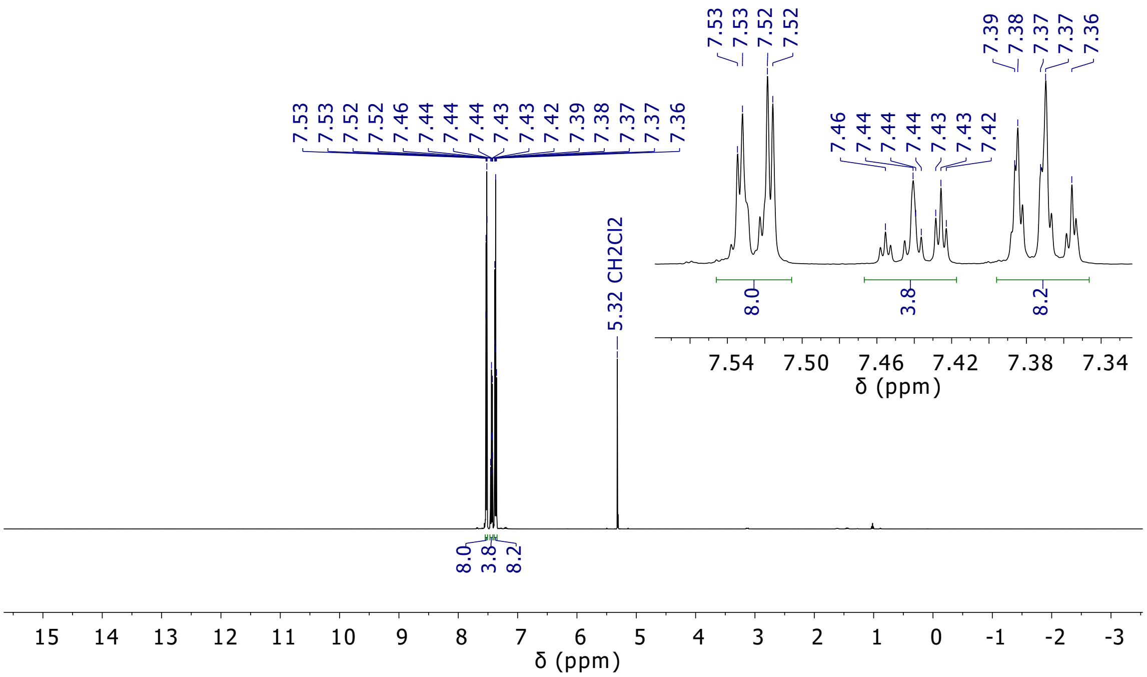

Figure S2. ^{1}H NMR spectrum of **1-Cl** (CD_2Cl_2, 500.2 MHz).

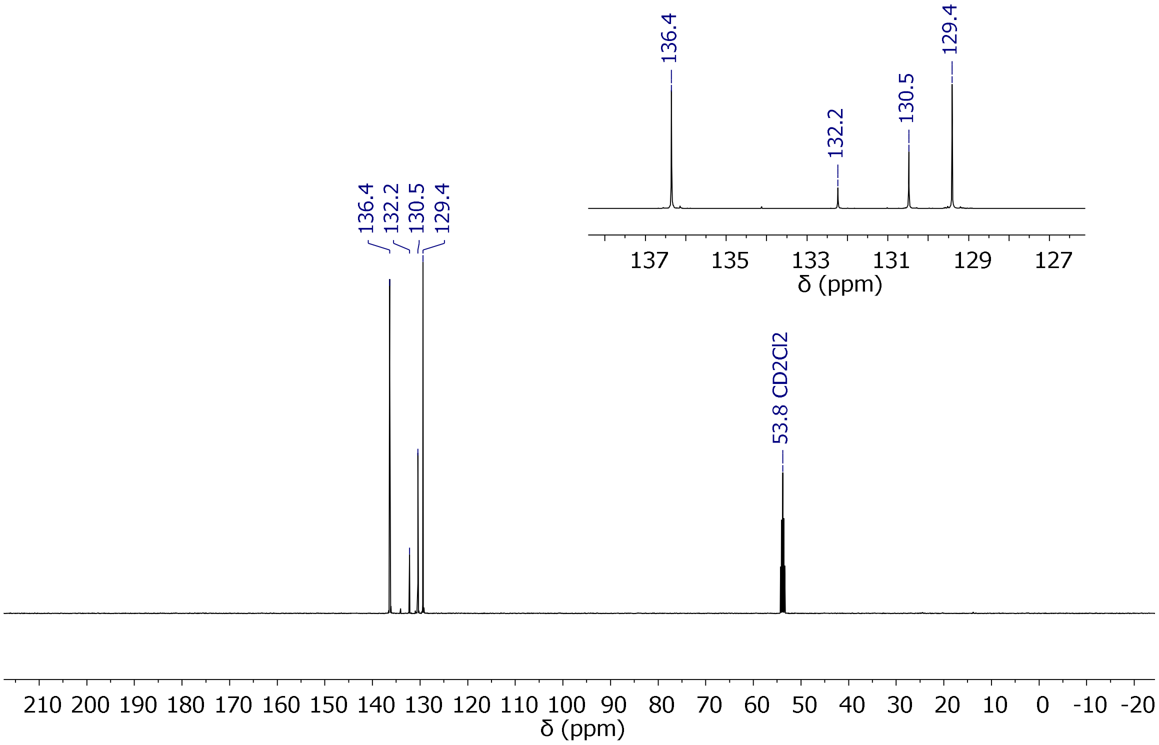

Figure S3. $^{13}C\{^{1}H\}$ NMR spectrum of **1-Cl** (CD_2Cl_2, 125.8 MHz).

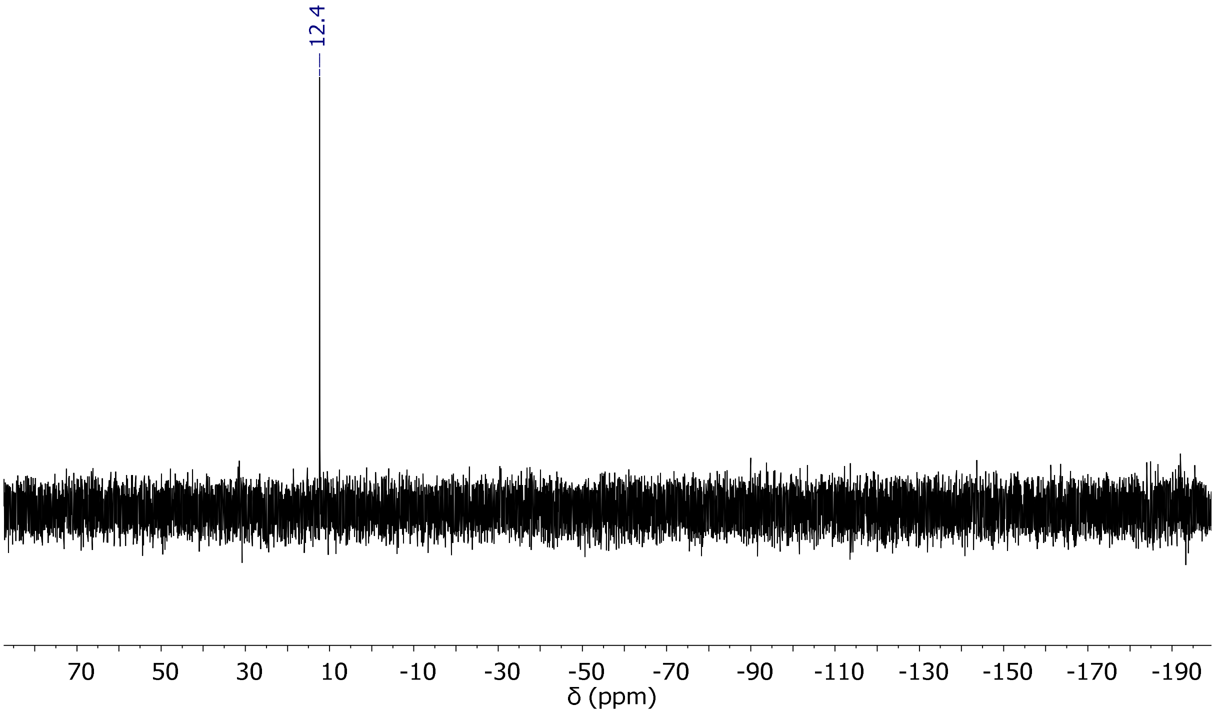

Figure S4. $^{29}Si\{^{1}H\}$ NMR spectrum of **1-Cl** (CD_2Cl_2, 99.4 MHz).

3.2. NMR Spectra of 2-Cl

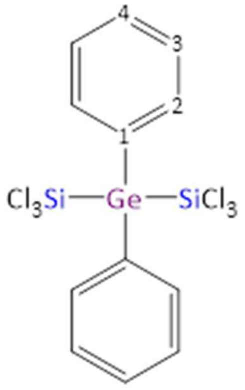

2-Cl

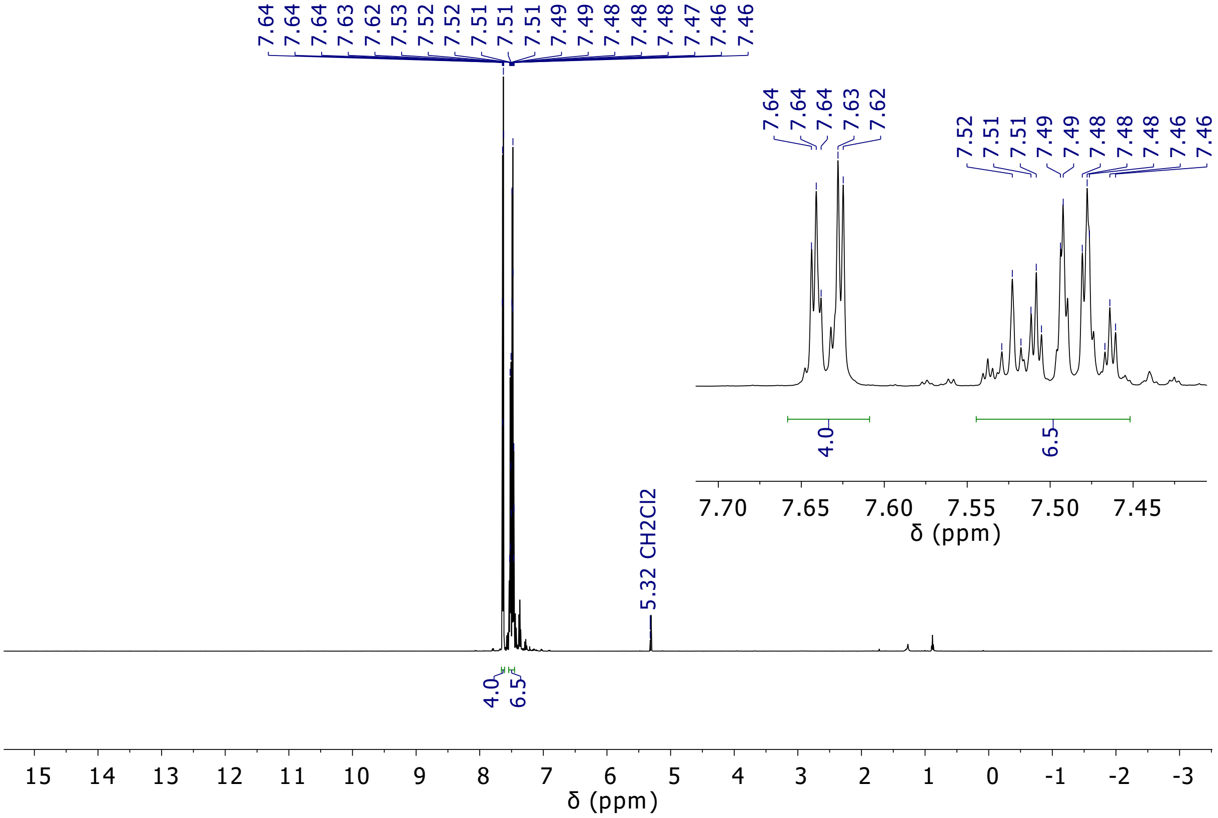

Figure S5. ^{1}H NMR spectrum of **2-Cl** (CD_2Cl_2, 500.2 MHz).

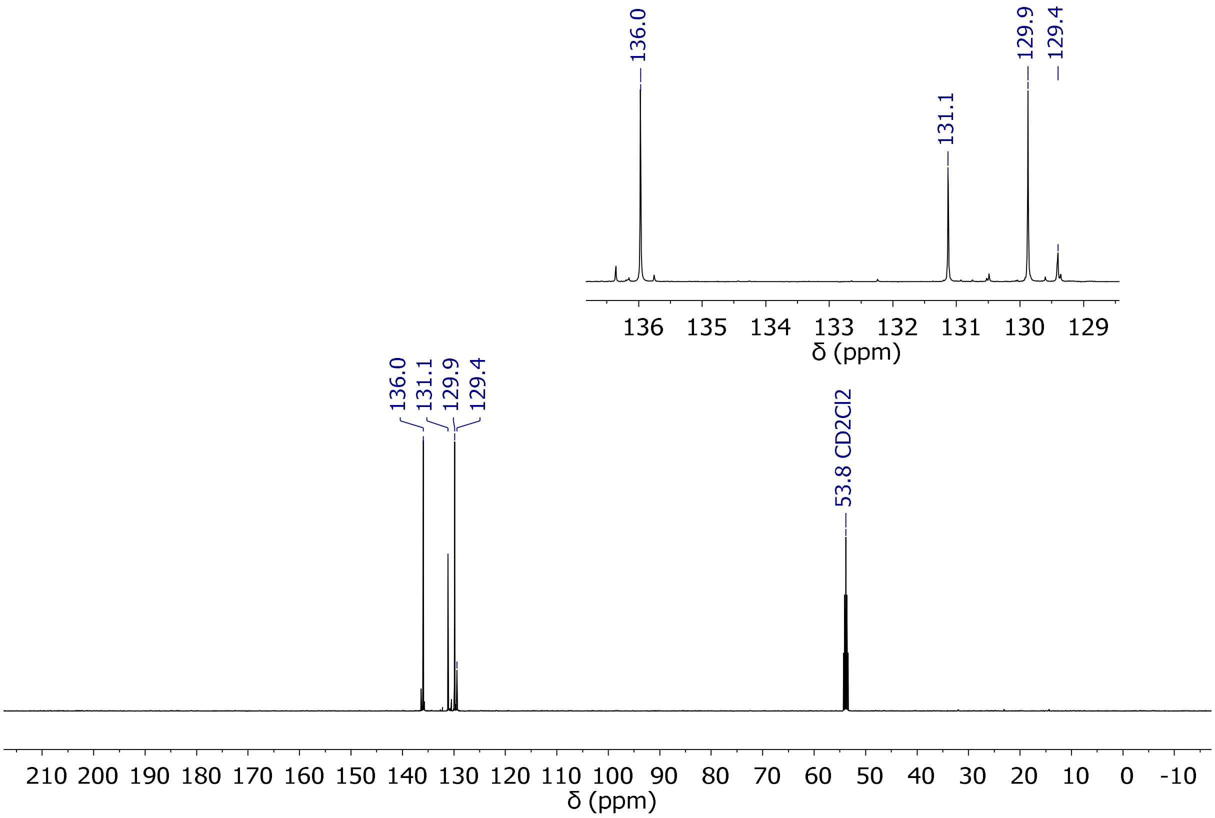

Figure S6. ${}^{13}C\{{}^{1}H\}$ NMR spectrum of **2-Cl** (CD_2Cl_2, 125.8 MHz).

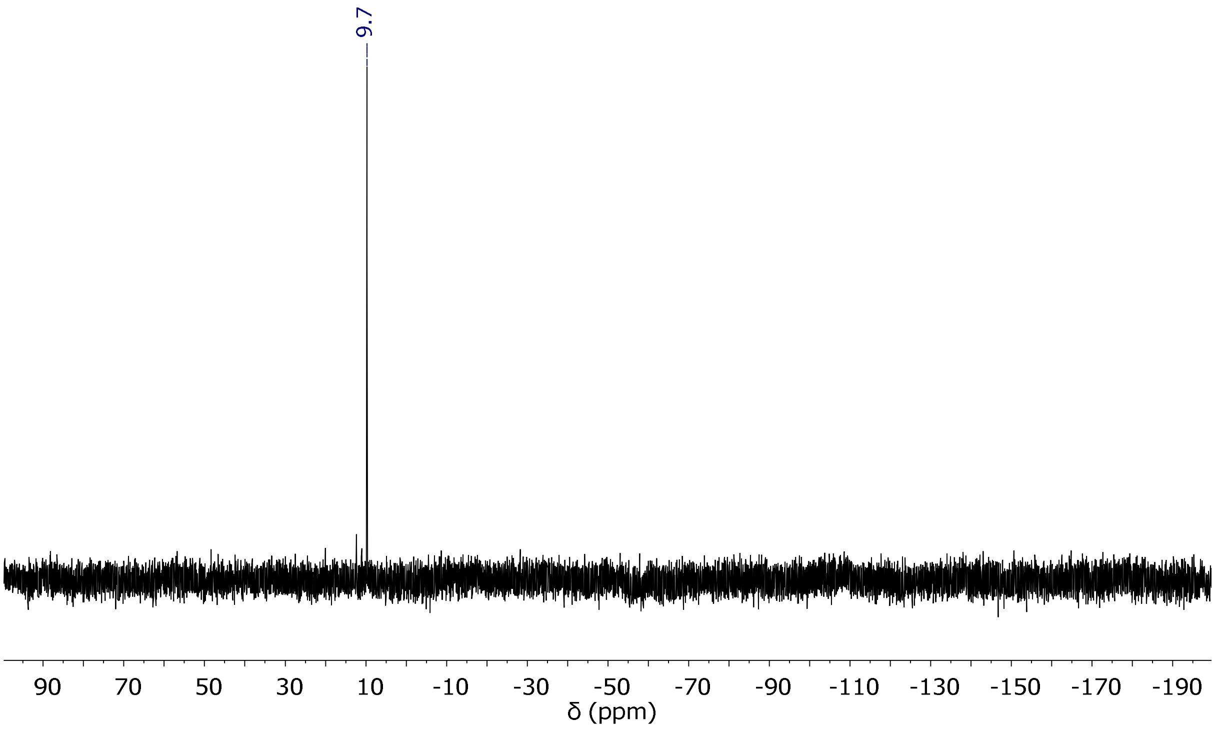

Figure S7. ${}^{29}Si\{{}^{1}H\}$ NMR spectrum of **2-Cl** (CD_2Cl_2, 99.4 MHz).

3.3. NMR Spectra of 3-Cl

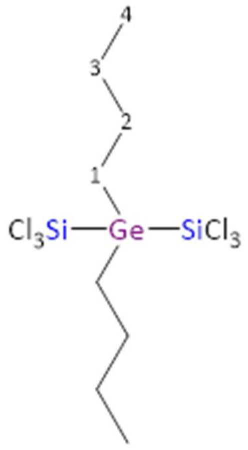

3-Cl

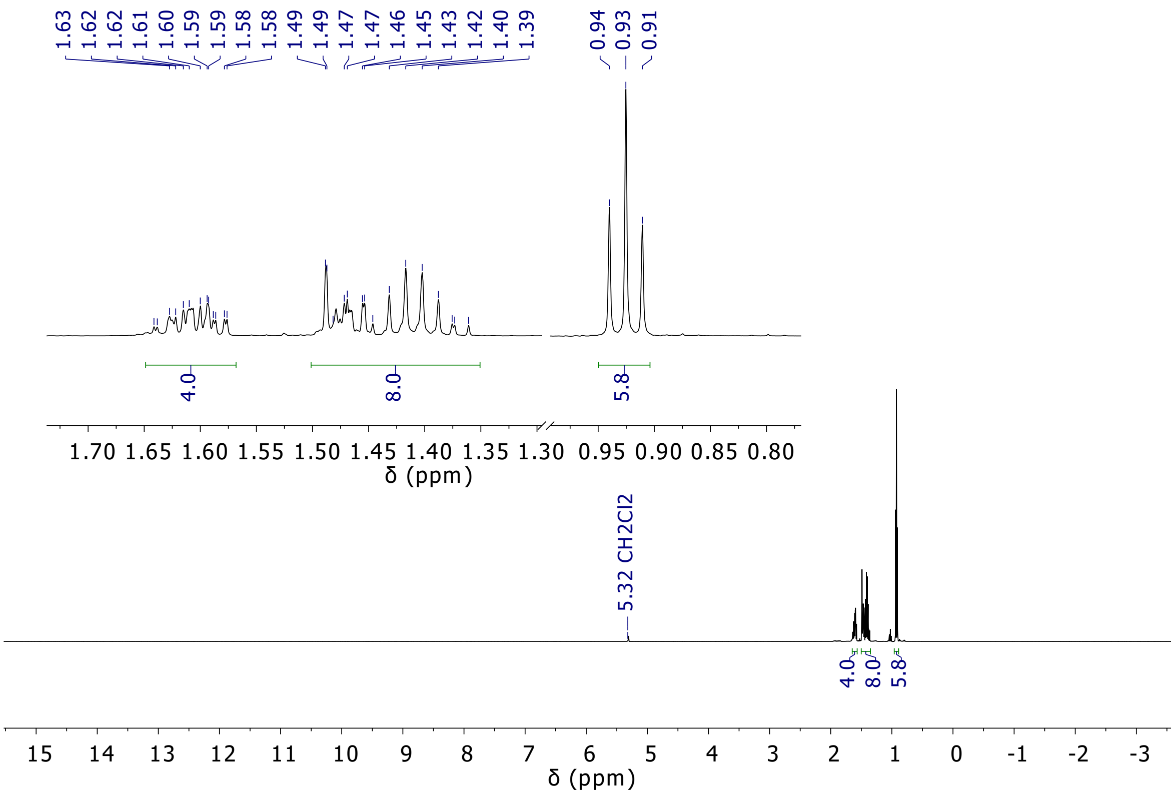

Figure S8. ^{1}H NMR spectrum of **3-Cl** (CD_2Cl_2, 500.2 MHz).

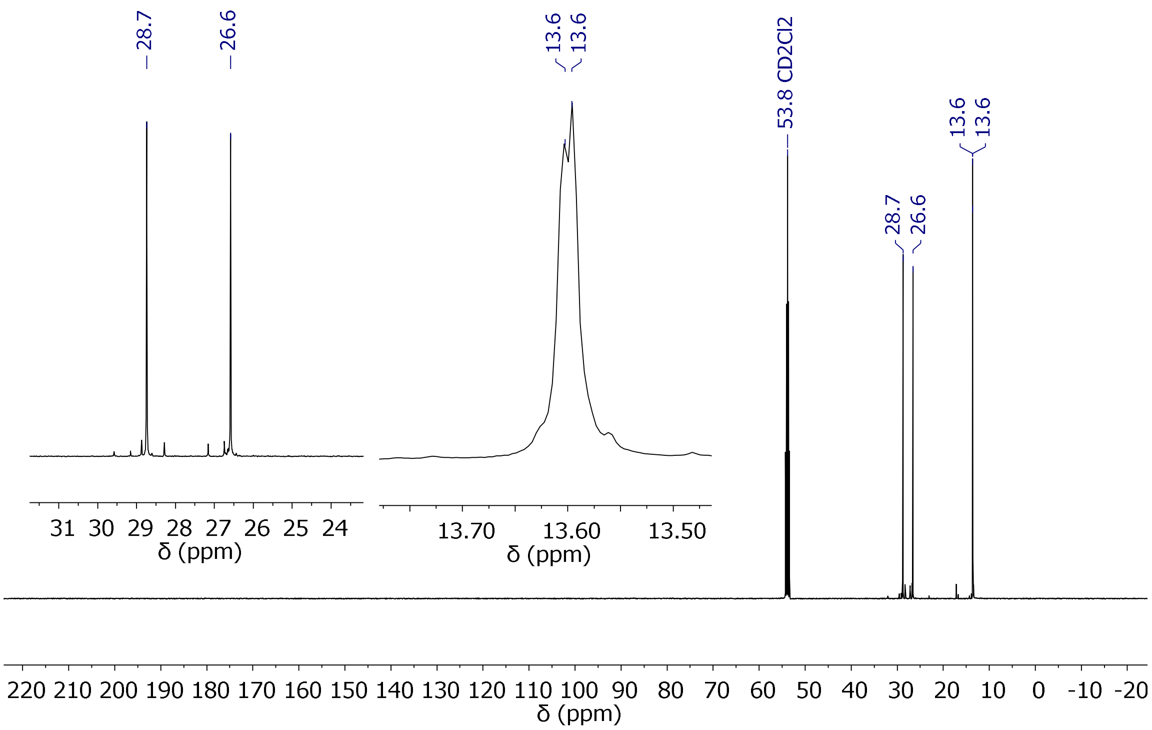

Figure S9. $^{13}C\{^{1}H\}$ NMR spectrum of **3-Cl** (CD_2Cl_2, 125.8 MHz).

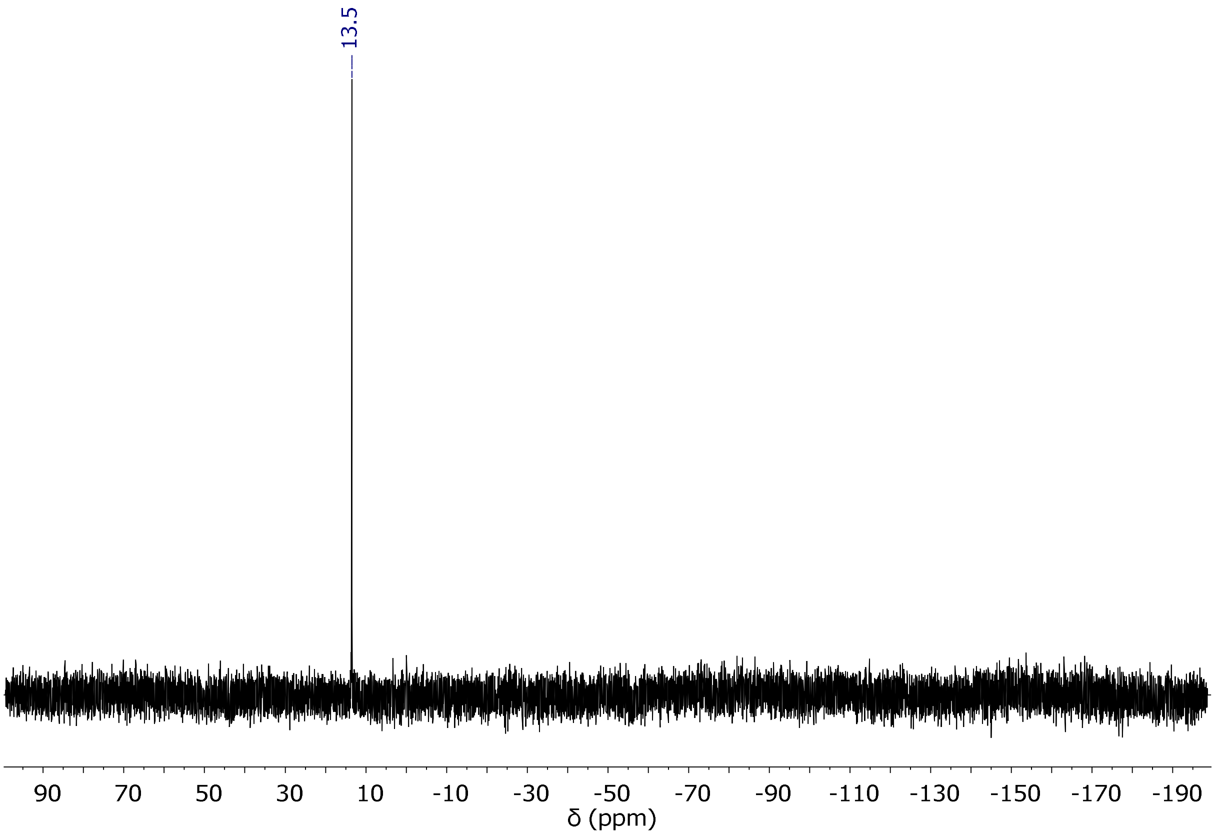

Figure S10. $^{29}Si\{^{1}H\}$ NMR spectrum of **3-Cl** (CD_2Cl_2, 99.4 MHz).

3.4. NMR Spectra of Cl–Ph2Ge–Ph2Ge–Cl

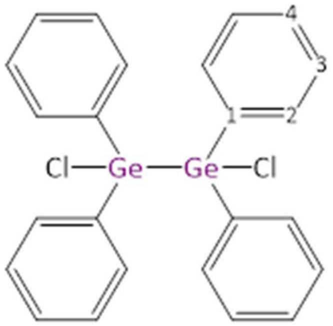

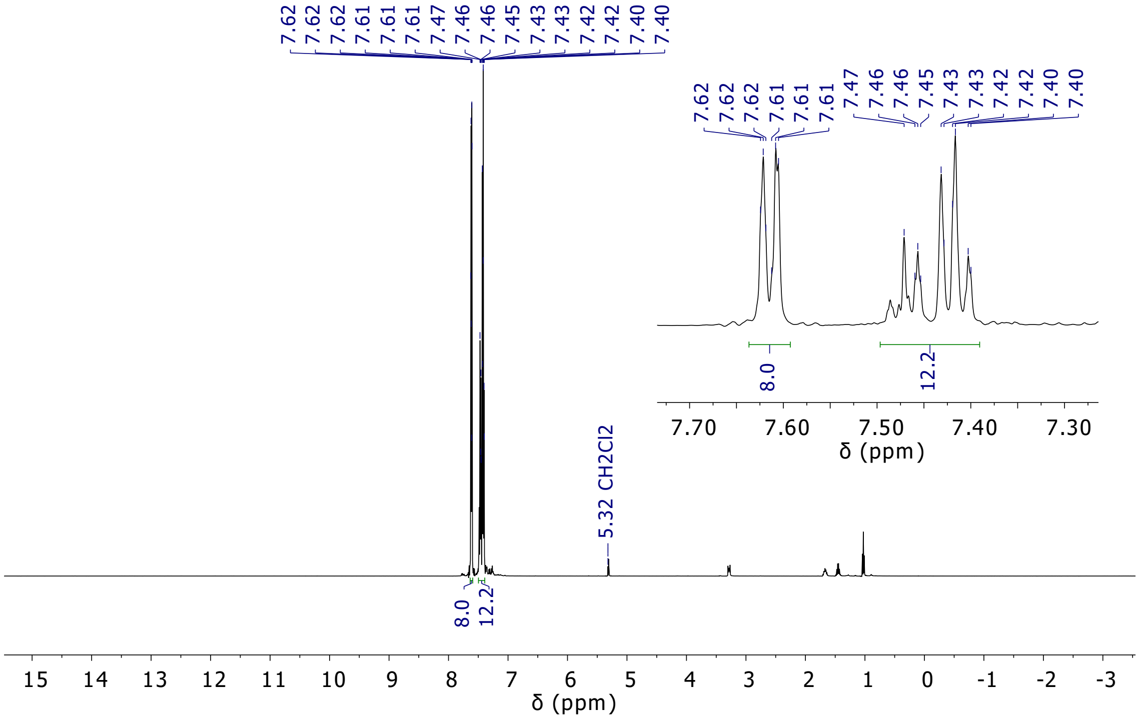

Figure S11. ^{1}H NMR spectrum (CD_2Cl_2, 500.2 MHz) of Cl–Ph2Ge–Ph2Ge–Cl containing small impurities of [*n*Bu4N]Cl (cf. $^{13}C\{^{1}H\}$ NMR spectrum).

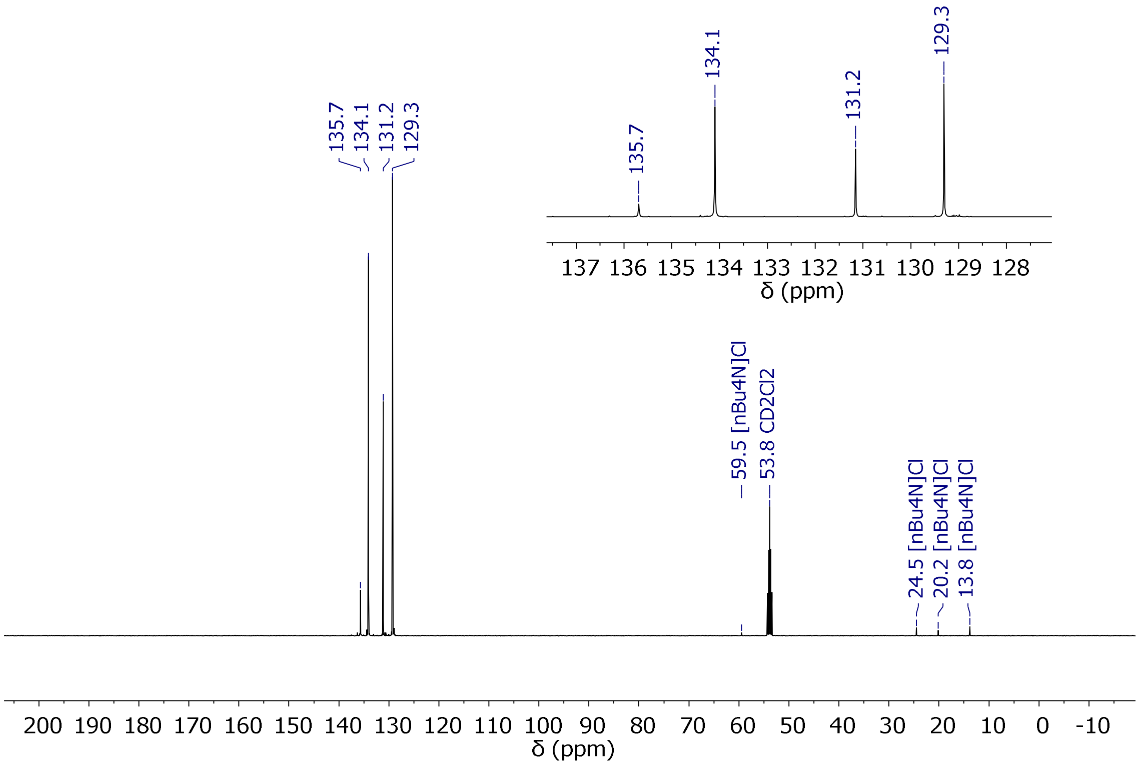

Figure S12. $^{13}C\{^{1}H\}$ NMR spectrum (CD_2Cl_2, 125.8 MHz) of Cl–Ph_2Ge–Ph_2Ge–Cl containing small impurities of [*n*Bu_4N]Cl.

3.5. NMR Spectra of 1-H

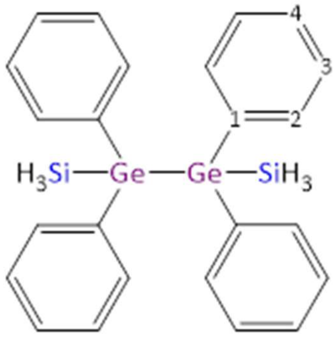

1-H

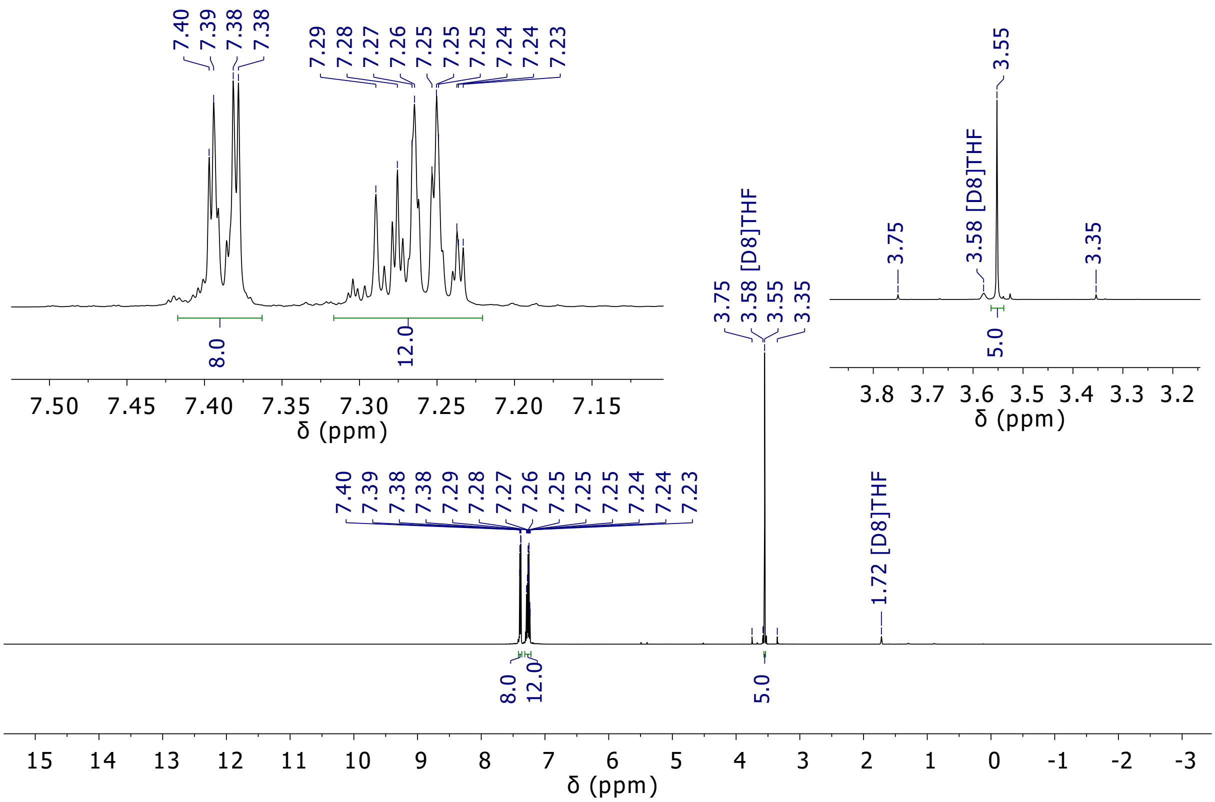

Figure S13. ^{1}H NMR spectrum of **1-H** ([D$_8$]THF, 500.2 MHz).

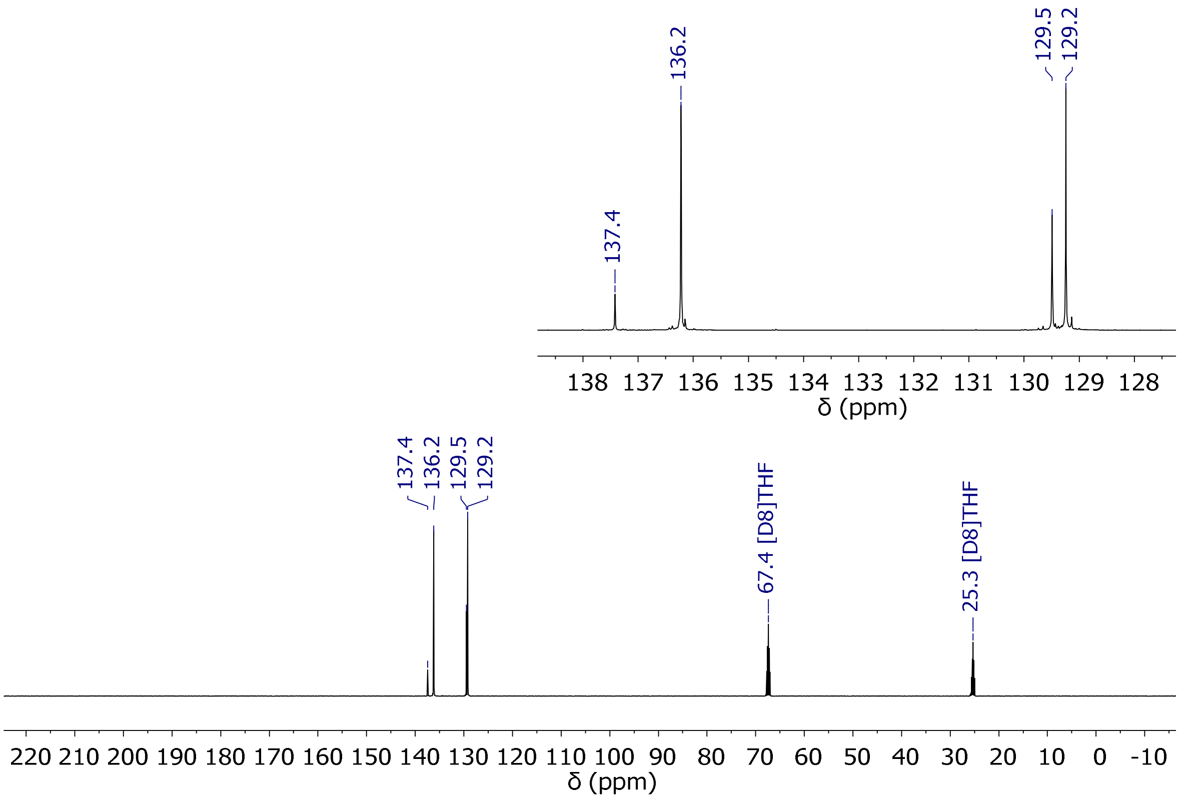

Figure S14. $^{13}C\{^{1}H\}$ NMR spectrum of **1-H** ([D$_8$]THF, 125.8 MHz).

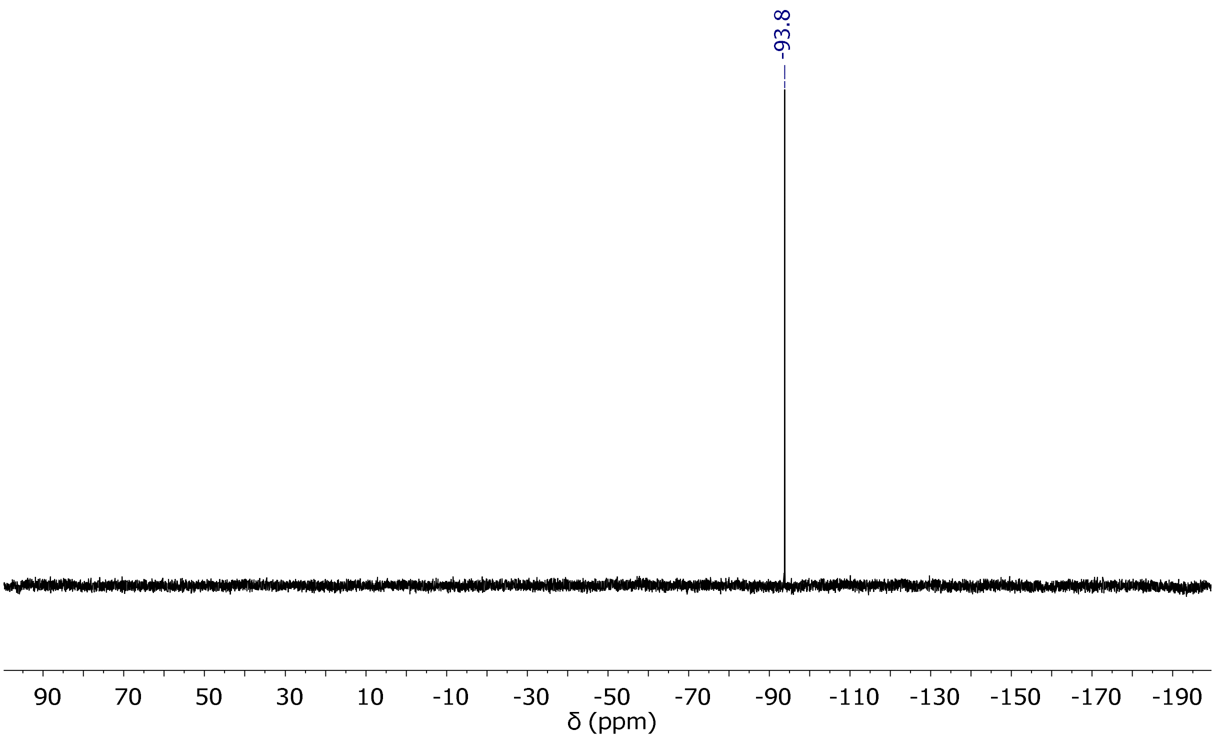

Figure S15. $^{29}Si\{^{1}H\}$ NMR spectrum of **1-H** ([D$_8$]THF, 99.4 MHz).

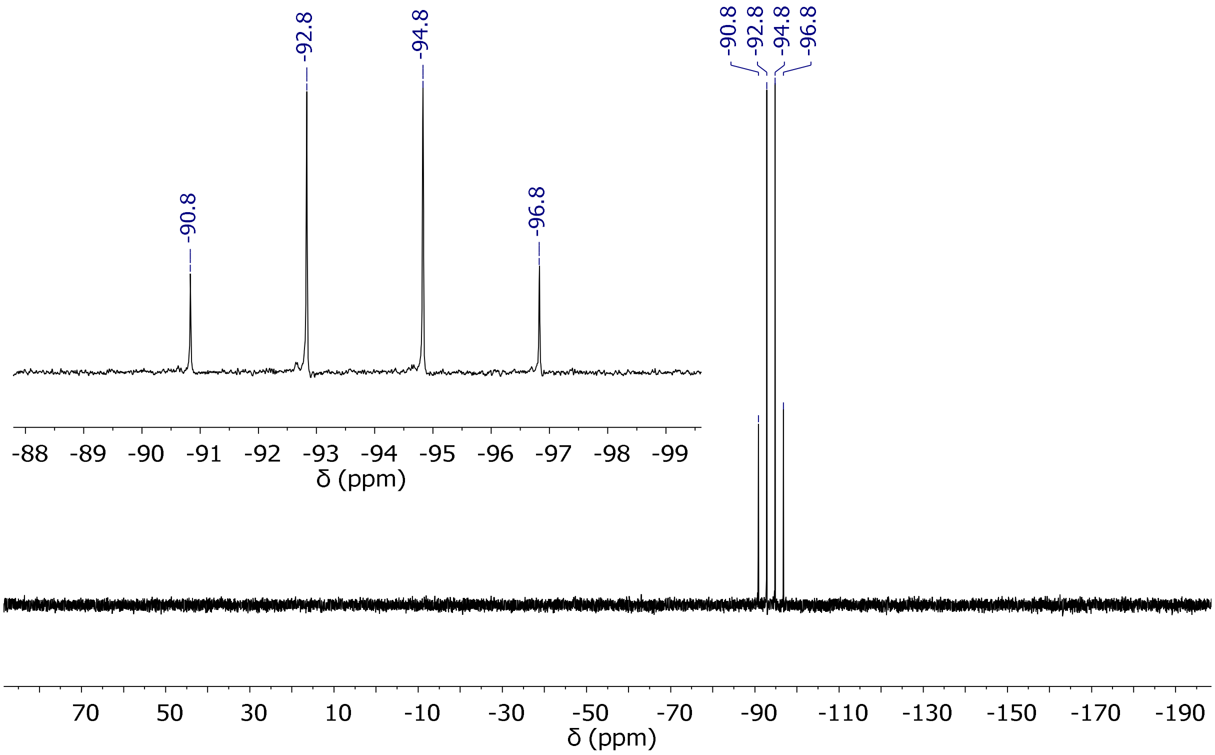

Figure S16. ^{29}Si NMR spectrum of **1-H** ([D$_8$]THF, 99.4 MHz).

3.6. NMR Spectra of 2-H

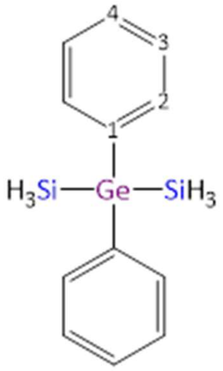

2-H

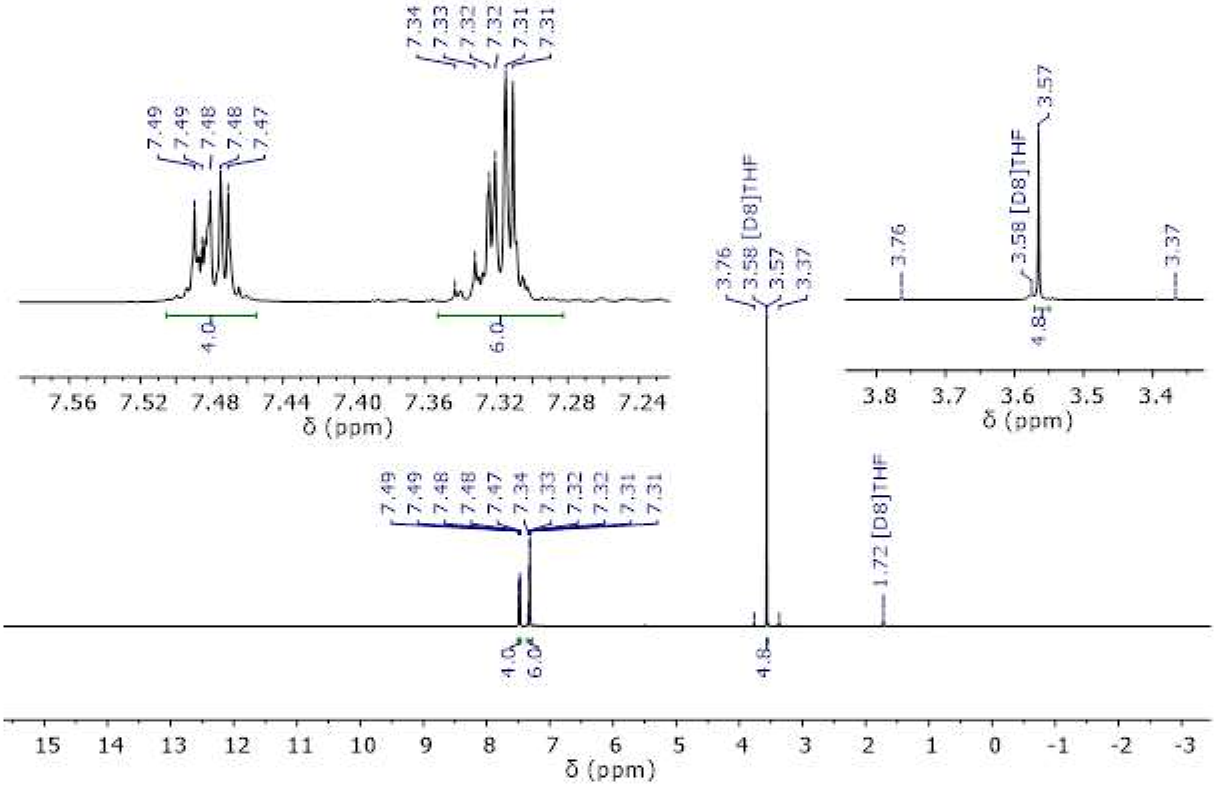

Figure S17. ^{1}H NMR spectrum of **2-H** ([D_8]THF, 500.2 MHz).

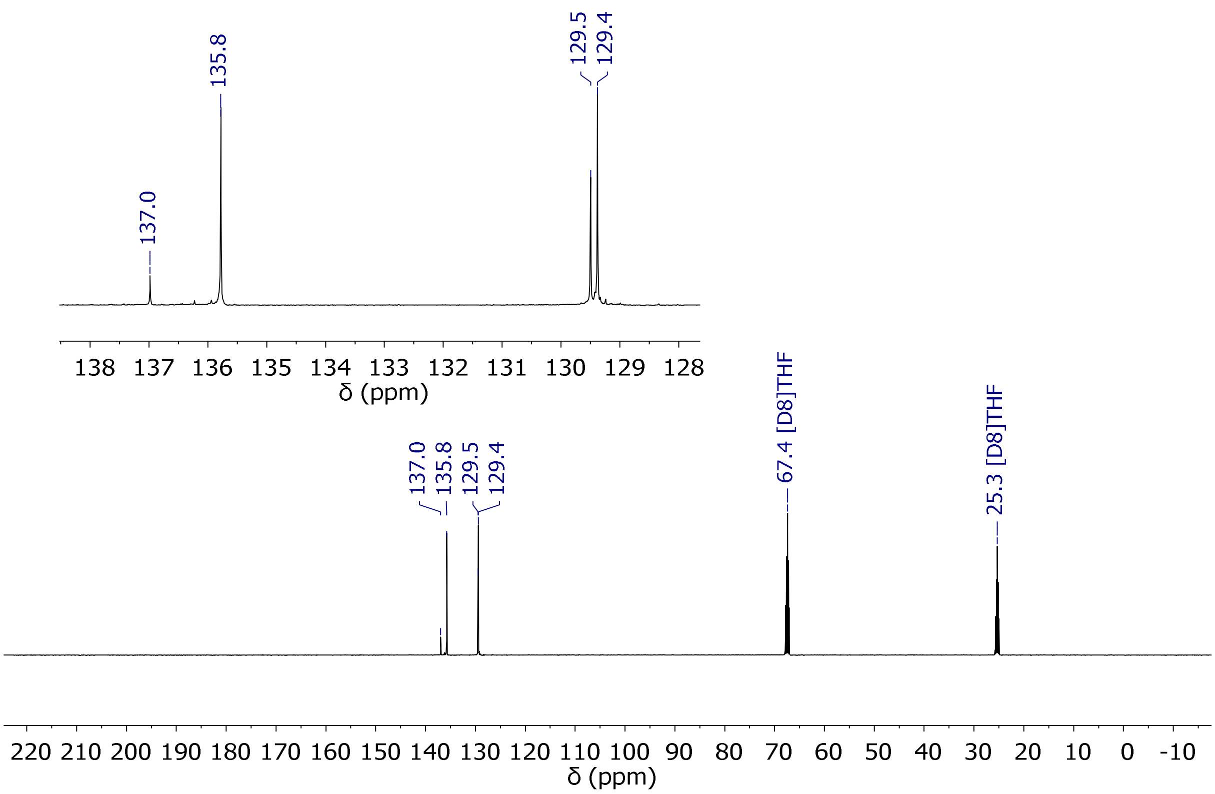

Figure S18. $^{13}C\{^{1}H\}$ NMR spectrum of **2-H** ([D8]THF, 125.8 MHz).

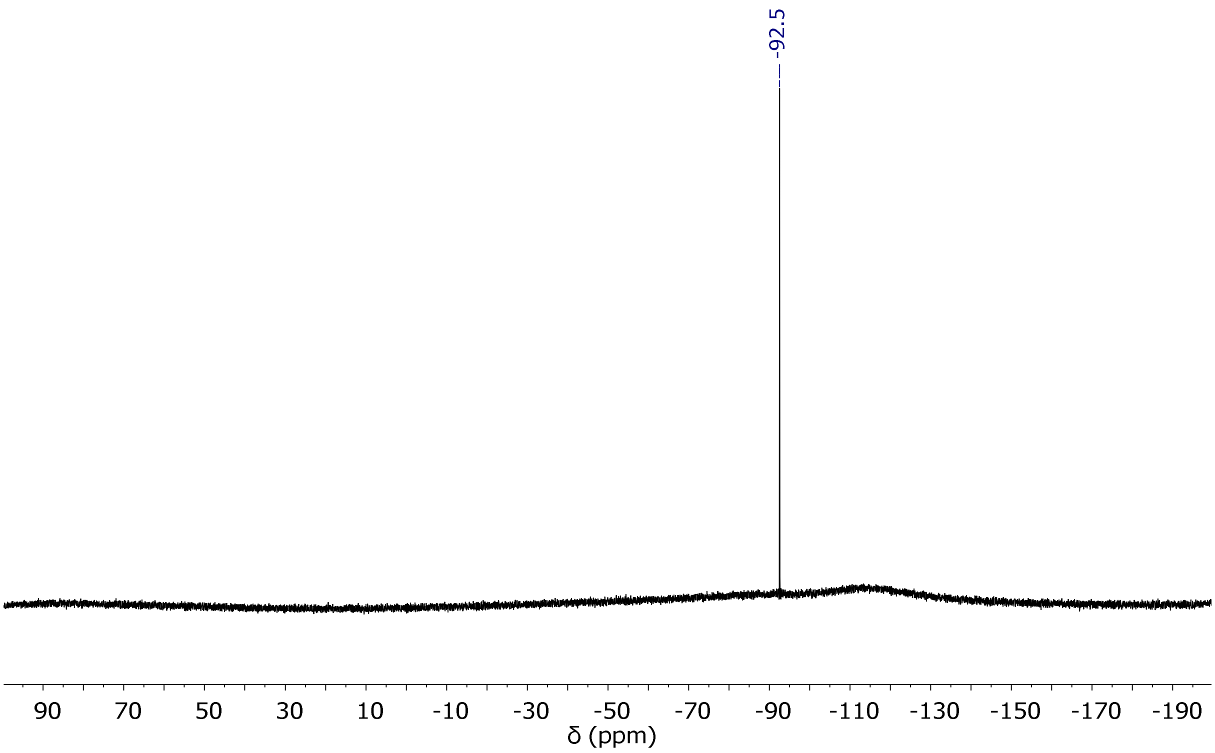

Figure S19. $^{29}Si\{^{1}H\}$+++ NMR spectrum of **2-H** ([D8]THF, 99.4 MHz).

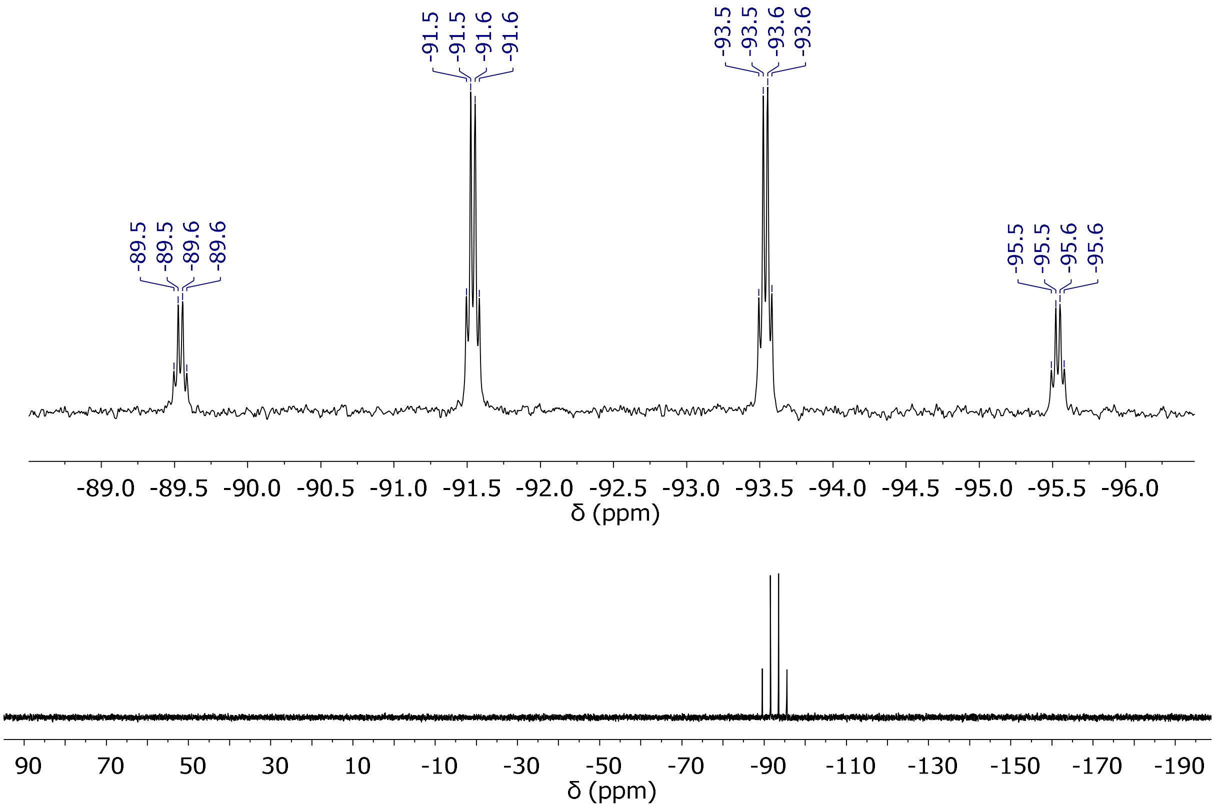

Figure S20. ^{29}Si NMR spectrum of **2-H** ([D$_8$]THF, 99.4 MHz).

3.7. NMR Spectra of 3-H

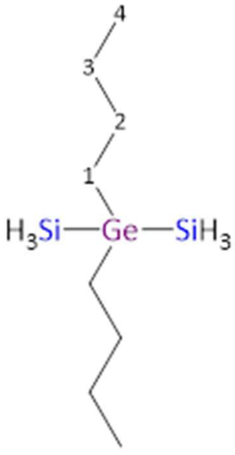

3-H

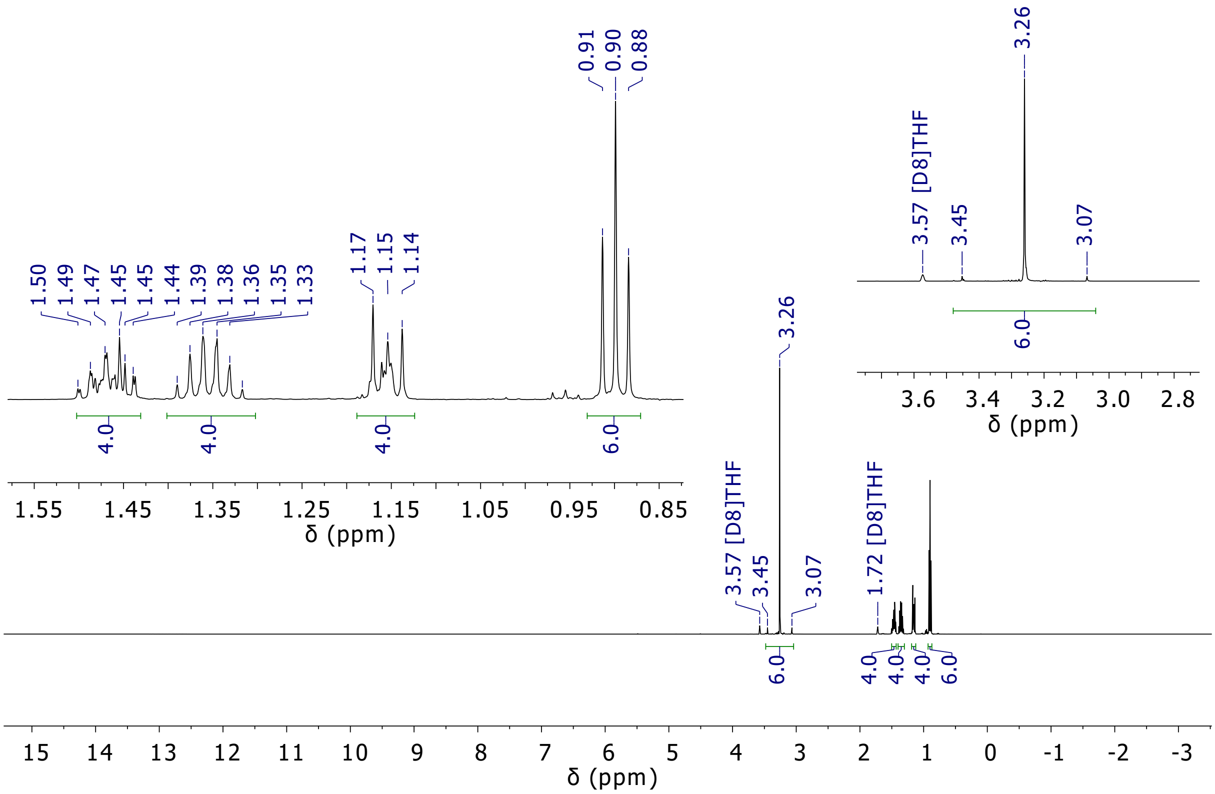

Figure S21. ^{1}H NMR spectrum of **3-H** ([D_8]THF, 500.2 MHz).

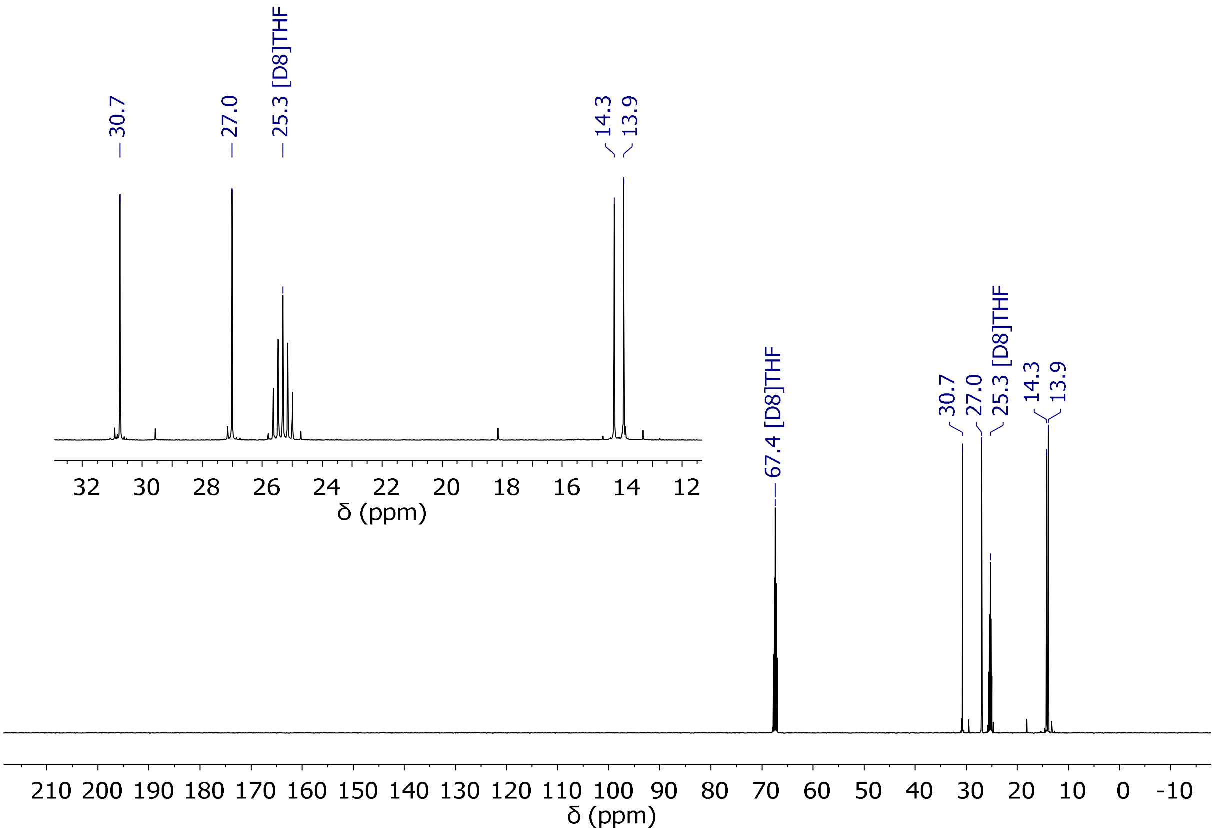

Figure S22. $^{13}C\{^1H\}$ NMR spectrum of **3-H** ([D8]THF, 125.8 MHz).

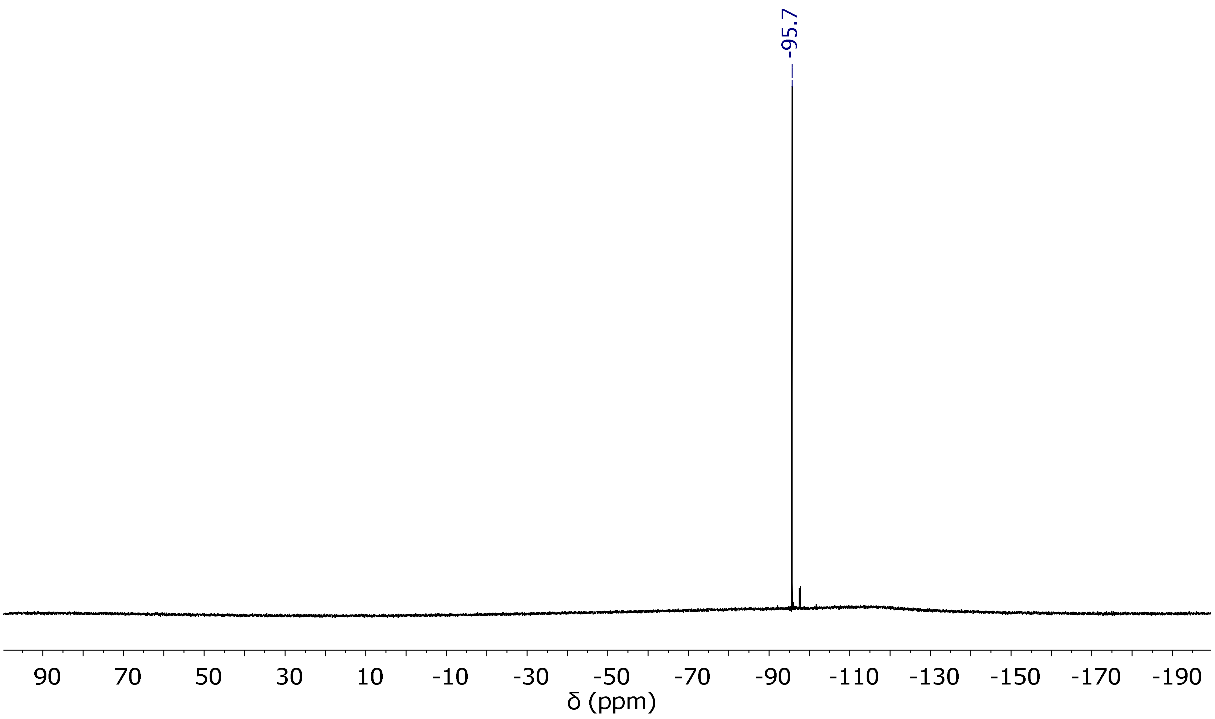

Figure S23. $^{29}Si\{^1H\}$ NMR spectrum of **3-H** ([D8]THF, 99.4 MHz).

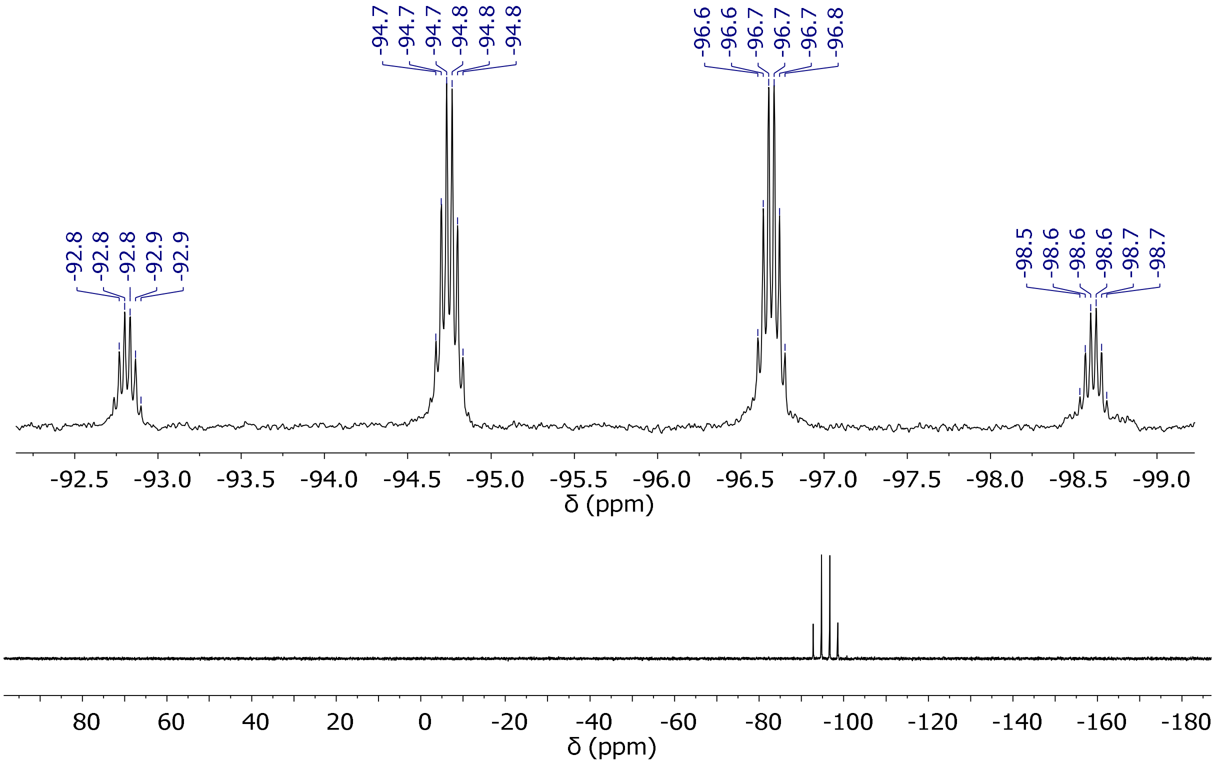

Figure S24. ^{29}Si NMR spectrum of **3-H** ([D$_8$]THF, 99.4 MHz).

4. Characterization of CVD Coatings

4.1. General Considerations

All reactions were carried out under an inert-gas atmosphere (dry argon or nitrogen) using standard Schlenk or glove-box techniques

(a)

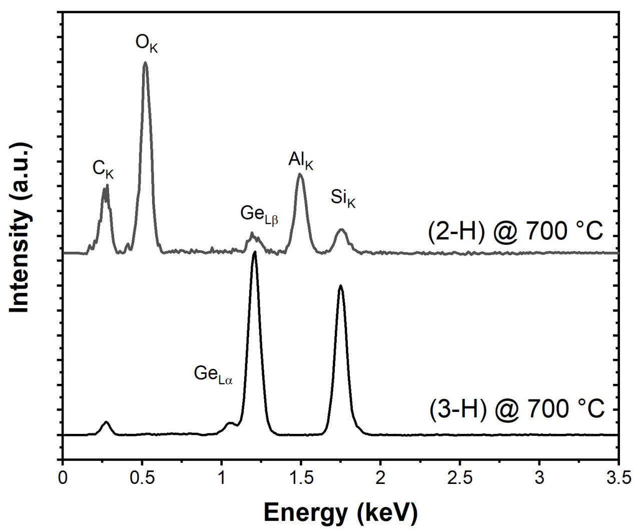

(b)

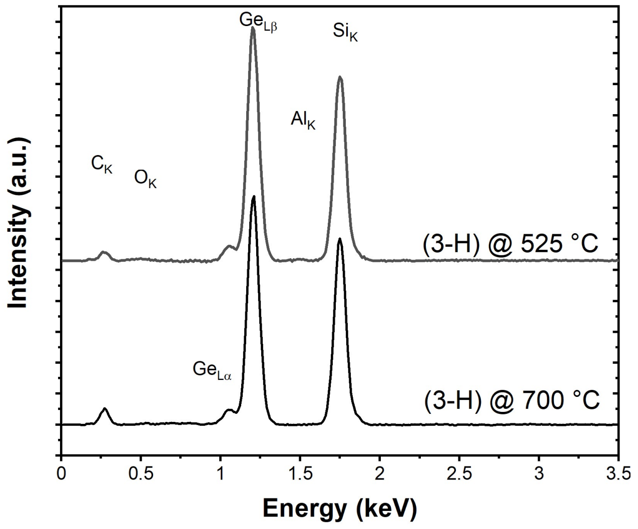

Figure S25. EDX spectra of (a) CVD films prepared using **2-H** and **3-H** at T_s=700 °C and (b) a comparison of the EDX spectra of $Si_{1-x}Ge_x$ films derived by using **3-H** at different substrate temperatures.

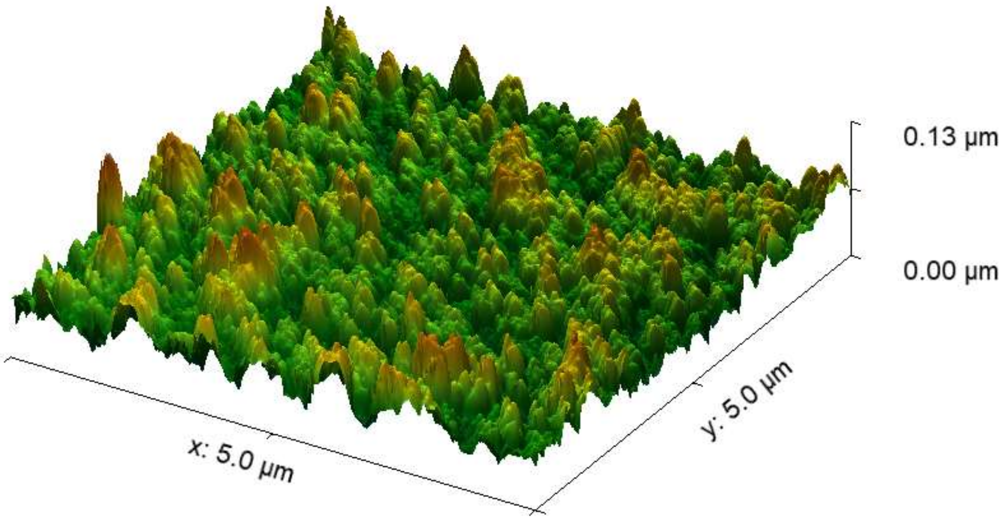

Figure S26. The AFM image of a $Si_{1-x}Ge_x$ deposit prepared at T_S=700 °C using **3-H** shows the formation of a smooth film without significant surface roughness.

(a)

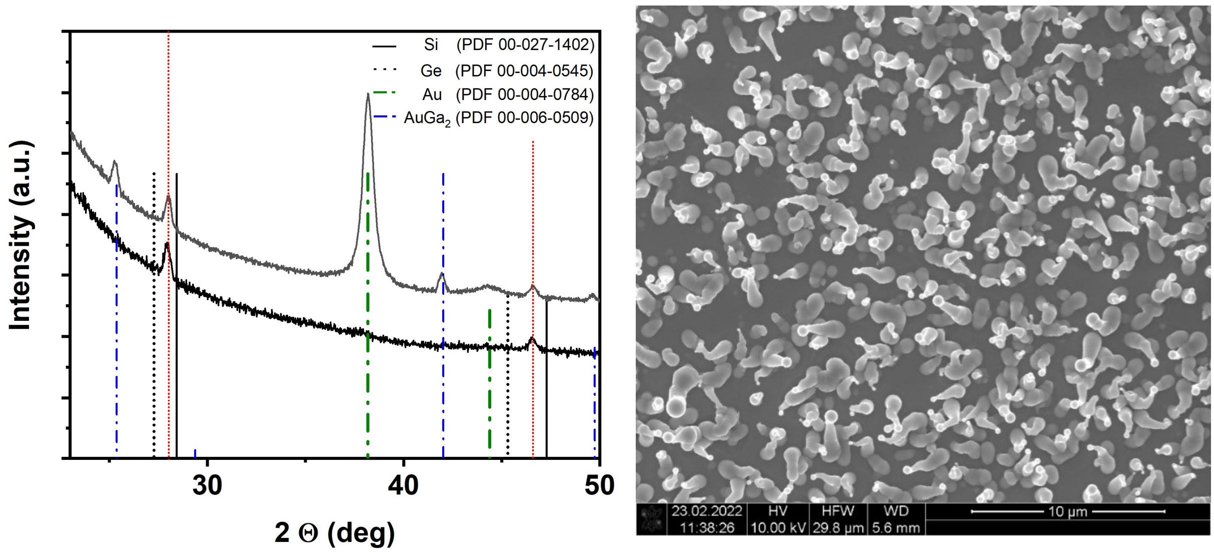

(b)

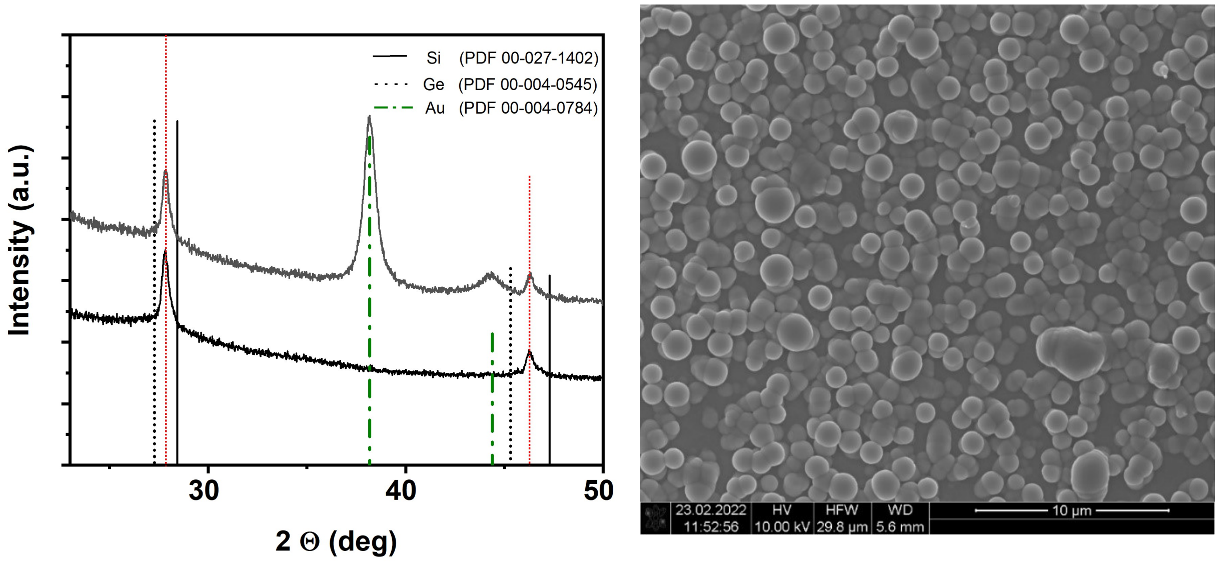

Figure S27. XRD and SEM images of Ga-supported crystallization of $Si_{1-x}Ge_x$ layers for (a) single Ga pulse and (b) three Ga pulses during the CVD using precursor **3-H** at T_S= 525 °C. Both films have been coated with Au post-growth to add a reference for XRD results. (a) represents Si:Ge ratio in the crystalline phase of 1.7 while in (b) merely 1.1 can be calculated by using Vegard's law.

5. References

1. Teichmann, J.; Kunkel, C.; Georg, I.; Moxter, M.; Santowski, T.; Bolte, M.; Lerner, H.-W.; Bade, S.; Wagner, M., Tris(trichlorosilyl)tetrelide Anions and a Comparative Study of Their Donor Qualities. *Chem. - Eur. J.* **2019,** *25*, 2740-2744.
2. Teichmann, J.; Wagner, M., Silicon chemistry in zero to three dimensions: from dichlorosilylene to silafullerane. *Chem. Commun.* **2018,** *54*, 1397-1412.
3. Satgé, J.; Rivière, P., Formation of the Germaniu-Germanium Bond by Reaction with Germyl-Metal Reagents. In *Inorganic Reactions and Methods*, J. J. Zuckerman; Hagen, A. P., Eds. VCH Publishers, Inc.: 1991; Vol. 9, pp 55-59.
4. Kraus, C. A.; Sherman, C. S., Preparation of Some Derivatives of Triphenylgermanium by Means of Sodium Triphenylgermanide. *J. Am. Chem. Soc.* **1933,** *55*, 4694-4697.
5. Cie, S. *X-AREA. Diffractometer control program system.*; Darmstadt, Germany, 2002.
6. Sheldrick, G., A short history of SHELX. *Acta Crystallogr., Sect. A* **2008,** *64*, 112-122.
